# Glial fibrillary acidic protein in Alzheimer’s disease: a narrative review

**DOI:** 10.1093/braincomms/fcae396

**Published:** 2024-11-07

**Authors:** Florine Leipp, Jérôme Vialaret, Pablo Mohaupt, Salomé Coppens, Aurore Jaffuel, Ann-Christin Niehoff, Sylvain Lehmann, Christophe Hirtz

**Affiliations:** Shimadzu France SAS France, Noisiel, France; IRMB-PPC, INM, Univ Montpellier, CHU Montpellier, INSERM CNRS, Montpellier, France; IRMB-PPC, INM, Univ Montpellier, CHU Montpellier, INSERM CNRS, Montpellier, France; IRMB-PPC, INM, Univ Montpellier, CHU Montpellier, INSERM CNRS, Montpellier, France; IRMB-PPC, INM, Univ Montpellier, CHU Montpellier, INSERM CNRS, Montpellier, France; Shimadzu Europa GmbH, Duisburg, Germany; Shimadzu Europa GmbH, Duisburg, Germany; IRMB-PPC, INM, Univ Montpellier, CHU Montpellier, INSERM CNRS, Montpellier, France; IRMB-PPC, INM, Univ Montpellier, CHU Montpellier, INSERM CNRS, Montpellier, France

**Keywords:** Alzheimer’s disease, glial fibrillary acidic protein, biomarker, astrocytes, reactive astrogliosis

## Abstract

Astrocytes are fundamental in neural functioning and homeostasis in the central nervous system. These cells respond to injuries and pathological conditions through astrogliosis, a reactive process associated with neurodegenerative diseases such as Alzheimer’s disease. This process is thought to begin in the early stages of these conditions. Glial fibrillary acidic protein (GFAP), a type III intermediate filament protein predominantly expressed in astrocytes, has emerged as a key biomarker for monitoring this response. During astrogliosis, GFAP is released into biofluids, making it a candidate for non-invasive diagnosis and tracking of neurodegenerative diseases. Growing evidence positions GFAP as a biomarker for Alzheimer’s disease with specificity and disease-correlation characteristics comparable to established clinical markers, such as Aβ peptides and phosphorylated tau protein. To improve diagnostic accuracy, particularly in the presence of confounders and comorbidities, incorporating a panel of biomarkers may be advantageous. This review will explore the potential of GFAP within such a panel, examining its role in early diagnosis, disease progression monitoring and its integration into clinical practice for Alzheimer’s disease management.

## Teaser

Leipp *et al*. provide a comprehensive review of glial fibrillary acidic protein (GFAP) as a blood-based biomarker for Alzheimer’s disease. This protein is a marker of astrocytic reactivity, a process associated with amyloid pathology. This review supports including GFAP in the diagnostic framework for Alzheimer’s disease.

## Graphical Abstract

**Graphical Abstract:**
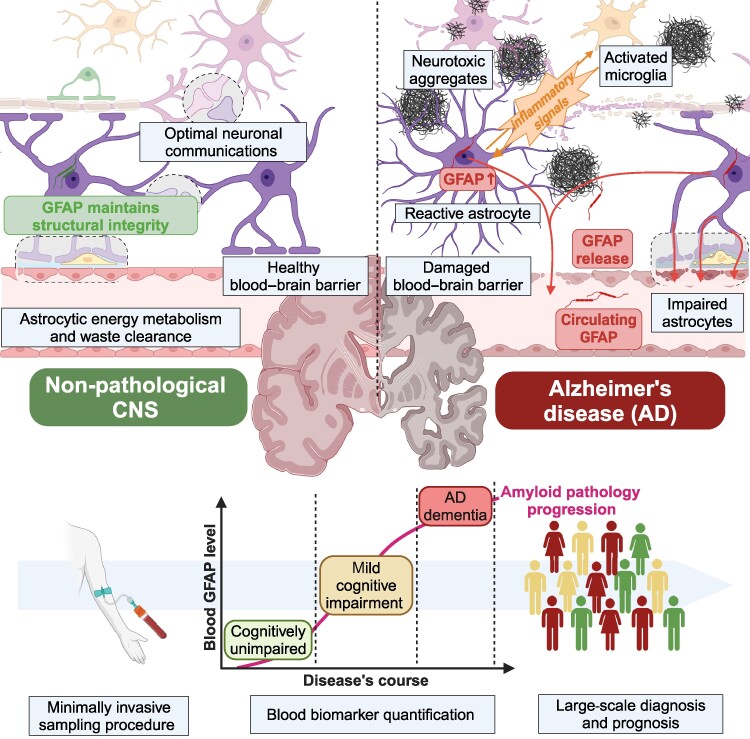

## Introduction

With a projected prevalence of over 152 million cases by 2050, Alzheimer’s disease is currently acknowledged as the prevalent form of dementia.^1^ This neurodegenerative disease leads to severe memory loss and cognitive impairment. Key pathological features of Alzheimer’s disease include intracellular neurofibrillary tangles of hyperphosphorylated tau (pTau) and extracellular senile plaques composed of amyloid-β peptides (Aβ) aggregates. These characteristic hallmarks are accompanied by glial response and chronic neuroinflammation.^2^ Efforts to develop targeted therapies for Alzheimer’s disease have consistently fallen short, leaving Alzheimer’s disease among the ranks of incurable conditions. Clinical trials targeting Aβ have shown low efficacy and significant side effects, resulting in an unfavourable risk-benefit ratio.^3^ The persistent challenge of finding treatments to prevent or slow down neurodegeneration without severe side effects highlights the multifaced nature of the disease’s pathogenesis. Consequently, research has shifted towards exploring and targeting alternative disease pathways.^4^ Despite the high accuracy of amyloid and tau-PET (positron emission tomography) imaging as well as cerebrospinal fluid (CSF) quantification of established biomarkers, such as pTau and Aβ, their limited availability, high cost and invasiveness underscore the need for alternative biomarkers. Plasma biomarkers for Alzheimer’s disease offer a timely and cost-effective alternative with less invasive sampling procedures.^5^ Recent developments in ultra-sensitive detection techniques have enabled the quantification of brain-derived proteins in blood, which reflect neurodegenerative processes. Among the extensively studied plasma biomarkers for Alzheimer’s disease, notable candidates include Aβ_1–42_, the Aβ_1–42_/Aβ_1–40_ ratio,^6^ tau proteins with phosphorylation at amino-acid positions 181, 217 and 231 (pTau-181, pTau-217 and pTau-231). These biomarkers reflect the pathological features of senile plaques and neurofibrillary tangles.^7^ Recently, novel blood biomarkers have emerged, including structural proteins from brain cells, such as neurofilament light chain (NfL) and glial fibrillary acidic protein (GFAP). NfL, a major constituent of axons, demonstrates a non-specific increase in response to brain injury or damage and correlates with neuroinflammation.^8^ GFAP, associated with astrogliosis, is linked to pathological processes in Alzheimer’s disease, particularly amyloid build-up, and neuroinflammation.^9^ The complexity and heterogeneity of neurodegenerative diseases complicate outcome prediction and patient care. The National Institute of Aging and Alzheimer’s Association (NIA-AA) framework characterizes Alzheimer’s disease as a biological construct, emphasizing diagnosis through its underlying pathological hallmarks and biomarkers. In light of recent advances, a proposed update to the 2018 [AT(*N*)] diagnosis criteria research framework document was presented at the Alzheimer’s Association International Conference (AAIC) 2023 held in Amsterdam.^10^ Among the major updates, the ATN framework now incorporates biomarkers linked to reactive astrogliosis, acknowledging its role in Alzheimer’s disease.^11^ In response to neurodegenerative conditions, astrocytes become reactive, undergoing morphological and functional changes.^12^ This includes GFAP overexpression and its release into the bloodstream.^13^ Reactive astrocytes produce inflammatory agents and are found surrounding amyloid plaques, suggesting their involvement in amyloid pathology and neuroinflammation.^14,15^ Recent studies suggest that astrogliosis occurs in the early stages of Alzheimer’s disease, potentially preceding established hallmarks of the disease, such as Aβ aggregation and abnormal tau protein accumulation.^16-18^ GFAP’s association with astrogliosis and emerging evidence of its involvement in the early stages of Alzheimer’s disease highlight its potential as a biomarker. Plasma GFAP has shown superior diagnostic accuracy compared with GFAP measured in CSF, effectively distinguishing Aβ-positive from Aβ-negative individuals, predicting disease in at-risk populations and differentiating Alzheimer’s disease from other neurodegenerative disorders such as frontotemporal dementia.^19-22^ Therefore, blood and CSF GFAP were added to the existing [AT(*N*)] framework as biomarkers for reactive astroglial response. Plasma biomarkers have demonstrated strong diagnostic and are now integrated with CSF and PET imaging biomarkers, serving as a powerful tool for large-scale screening and early diagnosis.^23^ In clinical practice, blood biomarkers provide significant advantages for diagnosing, prognosing and monitoring Alzheimer’s disease. They come with several advantages, including low invasiveness, simplicity in sample collection, affordability, speed of implementation and broad acceptance. This study explores the potential of GFAP as a blood-based biomarker for early Alzheimer’s disease diagnosis and monitoring of disease progression. By offering insights into GFAP’s role in reactive astrogliosis, we demonstrate its clinical relevance in enhancing diagnostic accuracy and advancing our understanding of neurodegenerative diseases.

### GFAP as a biomarker of reactive astrogliosis

#### Astrocytes functions

Astrocytes are the most prevalent glial cells in the central nervous system (CNS) and are involved in a broad diversity of physiological mechanisms, essential to ensure brain health. The cytoskeleton of astrocytes is composed of numerous intertwined fibrils called intermediate filaments, with GFAP being one of their major constituents.^24^ The endfeet of astrocytes processes surround blood vessels, providing structural integrity to the blood–brain barrier (BBB).^25^ Engaged in neurovascular coupling, they release signalling molecules to modulate vascular tone and provide proper oxygen and nutrient supply to activated neurons.^26^ Astrocytes also actively participate in energy metabolism, ensuring glucose uptake from blood vessels. They constitute the major site of glycogen storage in the CNS and contribute to glycolysis and lactate secretion, which are essential for neuronal energy supply.^27^ At the synaptic level, they contribute to neuronal communication and plasticity through specific ion channels, neurotransmitter transporters and receptors expressed on their surface.^28^ They modulate neurotransmitter release, maintain ion homeostasis and clear metabolites from the brain to prevent toxic accumulation.^29-31^ Aquaporin 4 (AQP4) is a water channel, localized in astrocytic membranes, facilitating water exchange between brain interstitial fluid and CSF, indirectly supporting the clearance of protein and metabolites from the brain.^32-34^ Beyond their role in metabolic processes, astrocytes also play a key role in developmental stages. They establish molecular boundaries, initiate synaptic maturation, axonal development and angiogenesis.^35,36^

Astrocytes divide into subtype populations that form clusters, each with different localization, function and molecular composition within the CNS. Protoplasmic astrocytes, predominantly found in the grey matter, have numerous and short processes that provide extensive contact with neurons, regulating extracellular neuronal microenvironment and contributing to synapse function and plasticity.^37^ Conversely, fibrous astrocytes, located in the white matter, have fewer but longer processes and are mainly involved in axonal myelination and metabolic support.^38^ However, recent findings shown that astrocytic heterogeneity is more complex than previously acknowledged reflecting their fine adaptation to local environments.^39^ Beyond morphological differences and spatial distributions, astrocyte subtypes exhibit transcriptomic and functional diversity across brain regions.^40^ Hasel *et al.*^41^ recently performed RNA sequencing and spatial transcriptomics to identify and characterize a marginal inflammatory astrocyte subtype that specifically expresses myocilin and genes involved in immune reactivity.^42^ These astrocytes are located at the brain surface beneath the pia mater and constitute the *glia limitans superficialis.*^41^ Additionally, other *glia limitans* astrocytes surround endothelial cells of the brain vasculature with their endfeet and constitute the *glial limitans vascularis*, providing mechanical support and maintaining the integrity of the BBB.^43^ These observations support the hypothesis that *glia limitans* astrocytes act as sentinels, poised to respond to inflammatory signals and may participate in the spread of the disease if disrupted.^44^ Further studies have identified other region-specific cluster populations of astrocytes across cortical layers.^45^ Their exact roles in health and disease remain unclear and require further investigation to understand regional susceptibility to diseases such as Alzheimer’s disease or Parkinson’s disease, in which astrocytes are extensively implicated.

Overall, astrocytes are multifaced indispensable components of the CNS, orchestrating a myriad of functions essential for neuronal health and synaptic connectivity while adapting to the needs of their local environment. Their intricate interactions with neurons and the vasculature underscore their pivotal role in maintaining brain homeostasis and supporting proper brain function throughout life.

#### GFAP structure

##### GFAP gene transcription and isoforms

**Figure 1:**
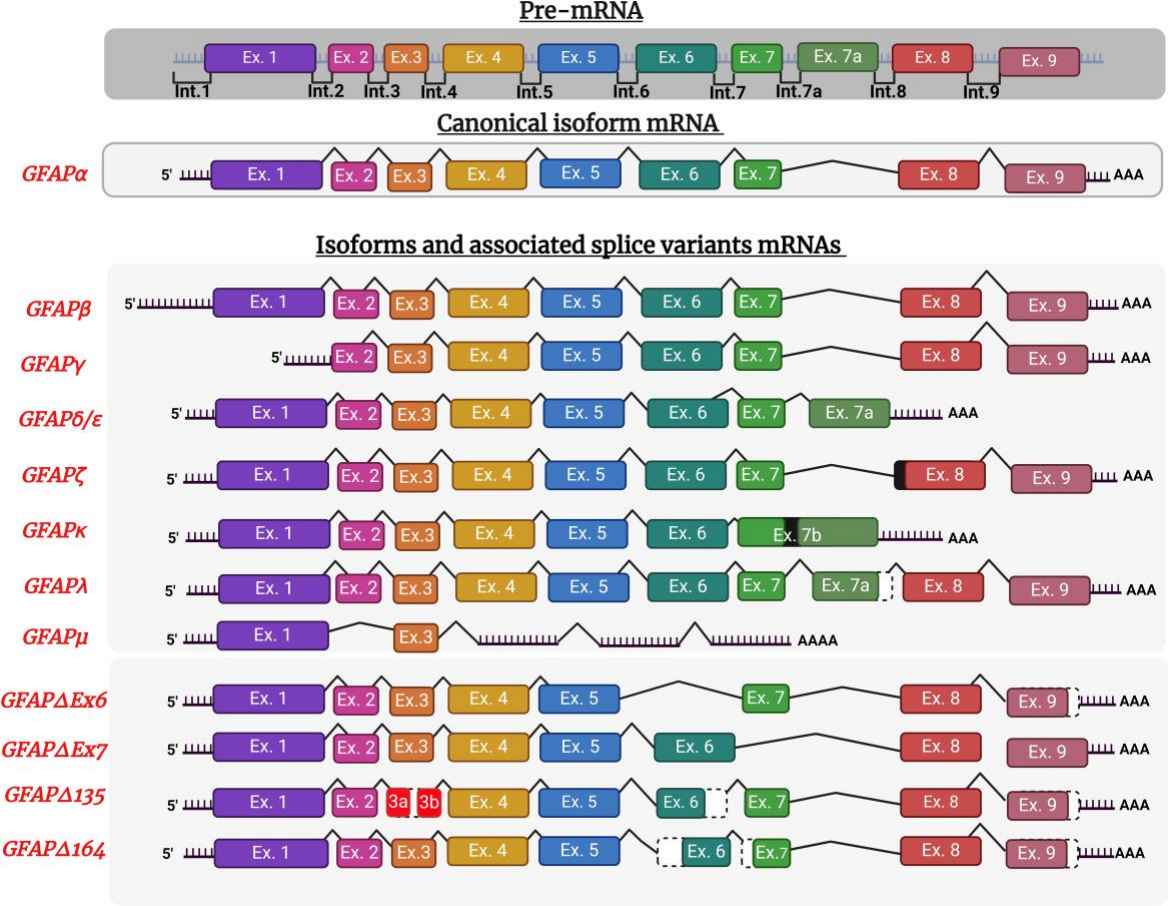
***GFAP* alternative splicing**. Pre-mRNA and isoform mRNAs of *GFAP* are represented in the figure. *GFAP* is the human gene encoding for human glial fibrillary acidic protein (GFAP) and contains nine exons presented in coloured boxes and separated by untranslated introns. Exons 1, 4 and 5 are constitutive for most isoforms (except GFAPµ), while the others undergo alternative splicing leading to 12 human identified isoforms. GFAPα is the canonical transcript and most abundant isoforms of GFAP. Exon 1 contains start codon ATG and encodes N-terminal domain of GFAP, except for GFAPβ where the transcription site is about 169 nucleotides upstream. Several isoforms lack exons. GFAPγ lacks exon 1 in the N-terminal domain, and GFAPµ transcripts contain only exons 1 and 3 lack most of the core coil domains exons. GFAPδ/ε and GFAPκ lack exons 8 and 9 encoding for the last coil and C-terminal domains. Conversely, splicing variant of several isoforms include additional exons like GFAPλ and GFAPδ/ε containing exon 7a after exon 7 and GFAPκ containing exon 7b containing both exon 7, intron 7a and exon 7a. GFAP +1 isoforms category gathers GFAPΔEx6, Δ7, Δ135 andΔ164 isoforms with exhibits several alternatives changes regarding canonical form. Among those, complete or partial deletion of exon 6, exon 7 or exon 3 and exon 9 shortening. Deletions are represented by black-dotted white boxes and intron transcription by black-filled boxes.

The human *GFAP* gene, located on chromosome 17q21, was identified through mouse, rat^46^ and human cDNA cloning studies.^47^ This gene comprises nine exons and eight introns, which are further diversified by the incorporation of four alternative exons and two alternative introns.^48^ Different protein isoforms are produced from the *GFAP* gene due to alternative splicing processes following RNA transcription.^49^ The complete sequence of *GFAP* was elucidated in the early 1990s using cDNA cloning experiments conducted on mouse brains, revealing a predicted full length of 432 amino acids with a molecular weight of 49.9 kDa.^50,51^ To date, 12 human proteoforms have been identified (Fig. 1).^49^ Among these, GFAPα is the predominant isoform, accounting for approximately 90% of total GFAP expression within the brain, spinal cord and peripheral nervous system (PNS).^52^ Further studies, utilizing RT-PCR assays, revealed that GFAPβ isoform was not only expressed in brain cells but also within Schwann cells of the PNS and in non-neural cells.^46,53,54^ Initially discovered in mouse brain and spleen, GFAPγ mRNA was subsequently also observed in human brain, spleen and bone marrow.^55^ GFAPβ transcription start site is 169 nucleotides upstream GFAPα putative start site. GFAPγ transcript lacks exon 1 and its transcription start site is thought to be 130 nucleotides before exon 2 starts, in intron 1.^55^ Furthermore, GFAPδ transcript was identified in rat cells,^56^ and its human homolog, GFAPε, was independently identified in a presenilin-binding partner study.^57^ GFAPδ/ε is preferentially expressed by neurogenic astrocytes of the subventricular region.^58,59^ GFAPδ mRNA includes the transcription of an additional exon (exon 7a) and exhibits a completely different C-tail, lacking exons 8 and 9.^56^ Additionally, GFAPζ with a longer transcript comprises the transcription of the last 284 nucleotides of intron 8 before exon 9 and was found to be extensively expressed in mouse brain.^60^ GFAPκ transcripts reported in mouse and pig cortex, cerebellum and striatum present an additional exon (exon 7b) before 8 exon which is composed of exon 7, intron 7a and exon 7a and exhibit a distinctive shortened and modified C-terminal tail.^61^ Although mRNAs were identified, there is currently no evidence for human protein expression of GFAPβ, GFAPγ, GFAPκ and GFAPζ yet.^60,62^ More recently, GFAPλ and GFAPμ transcripts were discovered within human brain and spinal cord. GFAPλ like GFAPδ contains exon 7a but keeps exons 8 and 9 transcriptions, while GFAPμ only contains exons 1 and 3 with a premature termination codon following exon 3.^63,64^ Moreover, GFAP has four alternative isoforms collectively termed GFAP + 1 present in astrocyte subgroups associated with single nucleotide frame-shift variants: GFAPΔEx6, GFAPΔ164, GFAPΔ135 and GFAPΔEx7.^65^ Increased levels of specific GFAP isoforms have been reported over the course of amyloid pathology. The work of Kamphuis *et al*.^66^ on Alzheimer’s disease brain donors revealed high expression of GFAPδ in reactive astrocytes surrounding plaques and spreading within the hippocampus where the disease severity is increased. In the same study, GFAP transcript levels for GFAPα, GFAPδ, GFAPζ, GFAPκ, GFAPΔ135 and GFAPΔEx7 were also found to be increased and correlated with brain amyloid build-up. Moreover, GFAP + 1 isoforms expression was observed in specific subtypes of non-reactive astrocytes, characterized by large cell bodies and long processes, with elevated expression in Alzheimer’s disease. This astrocyte population also responded to Aβ-related cellular stress *in vitro*. However, the differential effects of GFAP isoforms in astrogliosis remain largely underexplored, necessitating further investigations to clarify their implications.

##### GFAP protein structure and post-translational modifications (PTMs)

**Figure 2:**
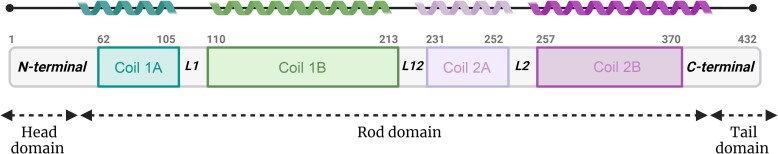
**Canonical GFAP 3D structure**. Canonical GFAP is a 432 amino-acid long protein composed of two core coil domains respectively divided into two subparts (**A** and **B**). Together, the four coil subparts structured in alpha helix constitutes the rod-domain situated between head and tail domains. The different coil parts are connected with three linkers (L1, L12 and L2).

The 3D structure of GFAP was elucidated through X-ray crystallography studies.^67^ It consists of four alpha-helical segments (1A, 1B, 2A and 2B) forming the ‘rod-domain’, surrounded by flexible non-helical N-terminal head and C-terminal tail domains (Fig. 2).^68^ Moreover, GFAP can undergo several post-translational modifications influencing its structure and properties.^69^ GFAP exhibits 16 phosphorylation sites with phosphorylation at serine 8 and 13 being involved in GFAP polymerization as well as the stability of intermediate filaments.^70,71^ Additionally, GFAP contains 28 arginine residues, which can undergo citrullination. Notably, this modification has been associated with several auto-immune or inflammatory conditions such as rheumatoid arthritis or multiple sclerosis, but not with Alzheimer’s disease.^72,73^ Furthermore, the single cysteine in the GFAP sequence might be subject to lipoxidation, potentially impacting the assembly of GFAP in the intermediate filament network.^74^

#### GFAP functions

##### GFAP ensures astrocytes’ structural integrity

**Figure 3:**
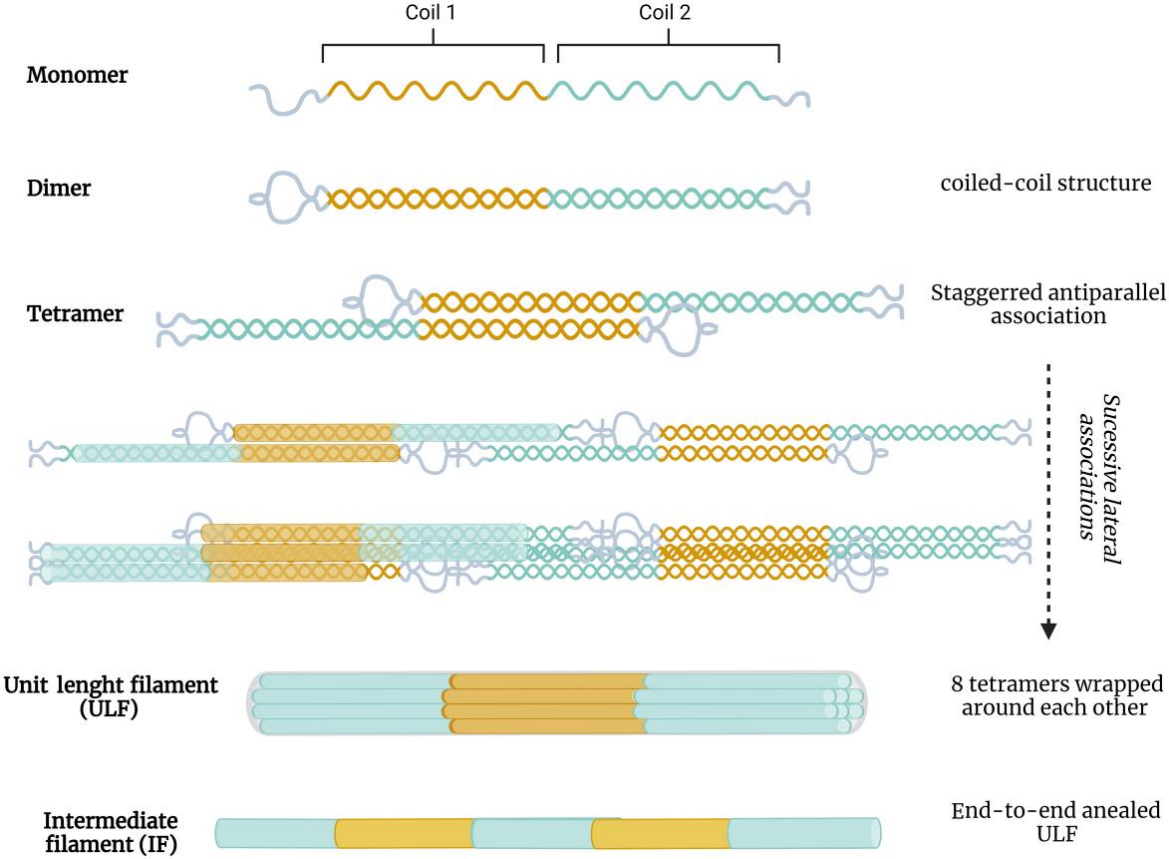
**From GFAP to intermediate filament**. Intermediate filaments are formed through the association of eight GFAP wrapped around tetramers generated from lateral association of coiled-coil structured GFAP dimers. Such dimers successively associate laterally into unit length filaments (ULFs) which are then connected end-to-end to form mature intermediate filaments (IFs).

GFAP was first identified on plaques with fibrous astrocytes and demyelinated axons obtained from the brains of individuals with multiple sclerosis.^24^ Along with vimentin, nestin and synemin, GFAP is one of the principal building blocks of intermediate filaments. Their specific localization in intermediate filaments was determined with immunostaining in cultured cells and tissues.^75^ The first step of intermediate filament assembly is the formation of parallel dimers in a coiled-coil structure, followed by the lateral antiparallel connection of dimers into tetramers and octamers. Subsequently, octamers associate laterally and are wrapped around each other to generate one unit length filament (ULF). Finally, ULFs associate into nanometer-long filaments to form mature filaments.^68,76^ Notably, GFAP can also form homodimers or heterodimers with vimentin.^77^ The entire formation process is presented in Fig. 3. As a type III intermediate filament protein, GFAP plays a crucial part in providing mechanical support, reinforcing astrocytes cytoskeleton and scaffolding for enzymes and organelles, as well as sensing mechanical cues from the extracellular environment.^78^

Over the past decades, several mouse models have been developed to explore GFAP’s extended physiological role. Gomi *et al*.^79^ reported that GFAP-null mice developed normally, showing no visible sign of deformity, infertility or developmental abnormalities compared with wild-type mice. This observation, corroborated by western blots in the same study, suggested that GFAP may not be essential for CNS development under physiological conditions. Although GFAP-null mice develop without visible abnormalities, GFAP is critical for maintaining CNS integrity, especially under conditions of aging or stress. For instance, other findings showed that aging GFAP-null mice (from 6 to 18 months) exhibited poorly vascularized and altered white matter due to impaired astrocytic scaffolding.^80^ Another study reported long-term deficits in CNS axon myelination and increased BBB permeability.^80^ More substantial deficiencies become evident following injury, especially head traumatism. One research group studied the consequences of simulated head concussion on sedated mice by subjecting them to weight drop impacts without head support.^81^ Among the 15 GFAP-null mice, 12 died primarily due to upper cervical spinal cord injury leading to respiratory arrest while all 14 wild-type mice survived. This suggests that GFAP within the intermediate filaments networks plays a role in maintaining the elasticity and structural strength of CNS. GFAP-deficient animals also exhibited increased sensitivity to cerebral ischaemia, with significant neuronal damage observed. Overall, these findings indicate that astrocytes provide brain protective roles, which are compromised under GFAP deficiency. GFAP interacts with various proteins and regulatory compounds, involved in intermediate filaments architecture and dynamics, including presenilin, LAMP-2A or αB-crystallin.^57,82,83^ Immunostaining experiments demonstrated that vimentin persists in the mature brain in the absence of GFAP, suggesting that vimentin compensates for the loss of GFAP in regions where it is typically expressed.^84^ Pekny *et al.*^85^ observed that GFAP(−/−) and vimentin(−/−) mouses still developed intermediate filaments, whereas GFAP(−/−) and vimentin(−/−) mice completely lacked intermediate filaments in their astrocytes cytoskeleton both *in vivo* and *in vitro.* These findings highlight vimentin’s ability to form intermediate filaments with either GFAP or nestin as a mandatory partner. However, nestin alone cannot assemble intermediate filaments, as corroborated by a subsequent study.^86^

GFAP expression is notably higher in fibrous astrocytes compared with protoplasmic astrocytes.^87^ Hasel *et al.*^41^ also reported that along with reactive gene expression, Myoc + astrocytes in the *glia limitans* exhibit elevated GFAP expression, surpassing baseline levels seen in other astrocyte subtypes. Furthermore, not all astrocytes express detectable levels of GFAP in healthy tissues. The elevated levels of GFAP in *glia limitans* astrocytes suggest that these cells may be ‘pre-reactive’, positioning them among the first responders to CNS insults and injuries.^31^

#### GFAP and reactive astrocytes

##### Astroglial gradual response to CNS injury

Astrocytes, beyond their diverse roles under normal conditions, respond to stress and injury signals of danger through astrogliosis.^12^ Initially, reactive astrogliosis is a protective process involving multiple cellular and molecular responses to CNS injury. Key participants include neuronal cells such as astrocytes, oligodendrocytes and neurons, as well as non-neuronal cells like microglia, pericytes and perivascular fibroblasts. Additionally, extrinsic cells infiltrating from the bloodstream such as leukocytes, platelets and fibrocytes can also contribute to this response.^88^ Collectively, these cells have the ability to affect and modulate each other’s activity through intricate molecular signalling pathways involving various neurotrophic factors and cytokines.^88^ Activated astrocytes regulate inflammatory response by controlling the entry, activation and trafficking control of peripheral immune cells such as leukocytes. Through direct cell-to-cell communication, astrocytes perform functions akin to those of antigen-presenting cells. Alternatively, they engage in indirect signalling by secreting soluble molecules, thereby actively contributing to the cerebral innate immune system.^89,90^ Astrogliosis is a heterogeneous phenomenon with its intensity and characteristics directly related to the severity of the damage. It ranges from mild astrogliosis, with reversible changes in gene expression and cellular hypertrophy, to severe astrogliosis, marked by enlarged glial scars and permanent tissue remodelling.^91^ An essential hallmark of astrogliosis is the overexpression of GFAP. Elevated GFAP levels correlate directly with the severity of neural damage, leading to structural hypertrophy of reactive astrocytes. In cases of severe and widespread brain injuries, astrocytes undergo extensive proliferation and form physical and chemical barriers surrounding lesion sites.^92,93^ This response is critical for containing damage and preventing its propagation to healthy tissues. The upregulation of GFAP acts as a marker of astrocyte activation following neural injury, underscoring their enhanced protective functions.

##### GFAP overexpression in astrogliosis

GFAP expression evolves during CNS maturation and aging. As previously described, vimentin is the major constituent of juvenile neuron tissue and is gradually replaced by GFAP during development.^94,95^ The onset of GFAP expression varies depending on the tissue under consideration. However, the number of cells expressing GFAP tends to rise with gestational age, particularly in the latter half of the gestational period.^96-98^ GFAP is first expressed in neural stem cells within the ventricular zone, later extending to the subventricular zone, where it persists postnatally and throughout adulthood.^99,100^ During aging, GFAP transcription and expression increase, primarily due to oxidative stress, a hallmark of normal aging.^101,102^ Nichols *et al.*^103^ reported increased transcriptional activity during aging, particularly in the hippocampus and frontal and temporal cortex regions. These areas are often impacted by neurodegenerative diseases associated with reactive astrogliosis. One of the primary functions of GFAP within the CNS is to provide structural support to astrocytes. Under pathological conditions such as inflammation, neurodegeneration or traumatic brain injury, GFAP expression is elevated leading to astrocyte changes in morphology through intermediate filament rearrangement. The effects of GFAP overexpression were investigated in transgenic mice carrying human genomic clones of the *GFAP* gene resulting in GFAP overexpression.^104^ The engineered mice presented hypertrophic astrocytes with intracellular intermediate filaments aggregates and inclusion bodies identical to Rosenthal fibres found in Alexander’s disease.^105,106^ GFAP overexpression, while essential for promoting tissue repair and containing damage in the CNS, can also lead to the formation of glial scars and disruption of neural circuits, ultimately contributing to cognitive decline. This dual role underscores the delicate balance between the protective and potentially harmful effects of astrocyte activation.^107^

##### Astrogliosis in Alzheimer’s disease

Astrocyte dysfunction is increasingly recognized as a key factor in the pathophysiology of Alzheimer’s disease and other neurodegenerative disorders, including Parkinson’s disease, multiple sclerosis, Alexander’s disease and Huntington’s disease.^108^ Emerging evidence suggests that reactive astrogliosis may precede classical pathological hallmarks such as Aβ deposition and abnormal tau protein aggregation.^109^ The astroglial response is consistently observed and progressively intensifies as Alzheimer’s disease progresses.^110^ This response leads to significant changes in astrocyte morphology and activity, profoundly impacting both disease severity and the healing process.^111^ Throughout neurodegenerative disease progression, reactive astrocytes have been reported to transition from a supportive role to acquiring a toxic role, with the degree of gliosis closely correlating with neurodegeneration severity. A GFAP immunoassay study on brain tissues revealed that the most pronounced glial response occurs in the hippocampus within the temporal lobe, the region where Alzheimer’s disease first induces neurological damage.^112^

Under pathological conditions, activated astrocytes upregulate the production of Aβ and release pro-inflammatory mediators, thereby initiating neuroinflammation and neurodegeneration.^113^ Accumulating Aβ, similar to alarmins, can attract astrocytes to the vicinity of the lesion through chemotactic molecules, affecting astrocytes morphology and functions and leading to progressive brain damage. Wyss-Coray *et al.*^114^ cultured mouse astrocytes and observed their progressive build-up around plaques in response to their release of monocyte chemoattractant protein-1. Their findings also indicated that astrocytes might degrade Aβ by binding to and phagocytosing Aβ deposits. Neuropathological findings further support the strong association between plaques and astrogliosis, with reactive astrocytes following the spatial distribution of Aβ plaques.^115^ Kamphuis *et al*.^60^ in line with previous observations by Xu *et al.*^116^ reported that GFAP-null astrocytes, unlike normal astrocytes, exhibited a delayed and reduced capacity to form tight and extensive boundaries around amyloid aggregates. These findings underscore the critical role of GFAP in facilitating effective confinement of neurodegenerative lesions through astrogliosis. In contrast to amyloid plaques, only few studies have examined the relationship between reactive astrocytes and tau tangles.^117^ It is thought that reactive astrocytes interact with tau protein to a lesser extent, predominantly in the later stages of Alzheimer’s disease. Astrocytes appear to abnormally internalize tau protein, leading to the formation of intracellular neurofibrillary aggregates that contribute to the propagation of pathological tau across the brain.^118^ Tau aggregates further induce astrocyte senescence with high mobility group box 1, a death-associated inflammation marker. It is also important to consider that throughout the progression of Alzheimer’s disease, Aβ and tau pathology experience a complex and intricate association.^119^ Nonetheless, the exact mechanisms underlying this relationship remain incompletely understood, and it is unclear whether this association exists independently of the amyloid plaques.^120^ Astrogliosis contributes to several mechanisms directly linked to neurodegeneration, exacerbating its pathological features. Pronounced reactive astrogliosis leads to the extensive production of reactive oxygen species (ROS), particularly hydrogen peroxide (H_2_O_2_). This increase in ROS contributes to the accumulation of pathological tau, cerebral atrophy and cognitive decline through oxidative stress.^121^ Neuronal hyperexcitability, characterized by an abnormal increase in neuronal activity, is another pathological feature in neurodegeneration. This hyperexcitability, potentially resulting from impaired ion channel function, synaptic irregularities or neurotransmitter imbalances is closely associated with neuronal death and cognitive decline.^122^

Neuroinflammation initially serves as a protective response, defending the brain against potential threats and counteracting disease progression. However, as Alzheimer’s disease advances, chronic inflammation persists and exacerbates neurodegenerative damage.^123^ This process involves a complex multicellular network, including microglia, astrocytes, oligodendrocytes and neurons, which interact through a cascade of signalling pathways that ultimately amplify inflammation and contribute to neuronal damage.^88^ Following Aβ deposition, activated microglia and reactive astrocytes engage in tightly regulated communication, significantly contributing to the neuroinflammatory response. This interaction is a hallmark of Alzheimer’s disease’s progression and highlights the pivotal role of these glial cells in sustaining inflammation. A major factor sustaining this neuroinflammation is the dysregulation of calcium signalling, which establishes a vicious cycle that perpetuates glial activation and drives neurodegeneration.^124^ Elevated levels of calcineurin in astrocytes and microglia induce the production of pro-inflammatory factors such as cytokines including interleukins, tumour necrosis factor-α and ROS. These factors further disrupt Ca^2+^ pathways, leading to progressive neuronal loss and subsequent cognitive impairment.^125^ In 5xFAC, an Aβ-bearing mouse model, calcineurin hyperactivation impaired astrocytic glutamate transporter function, causing deficient glutamate buffering, which is known to have excitotoxic effects.^126^ Additionally, oligodendrocytes exposed to these inflammatory signals exhibit impaired survival and function, disrupting myelination and exacerbating white matter damage, further contributing to cognitive decline.^127^

**Figure 4:**
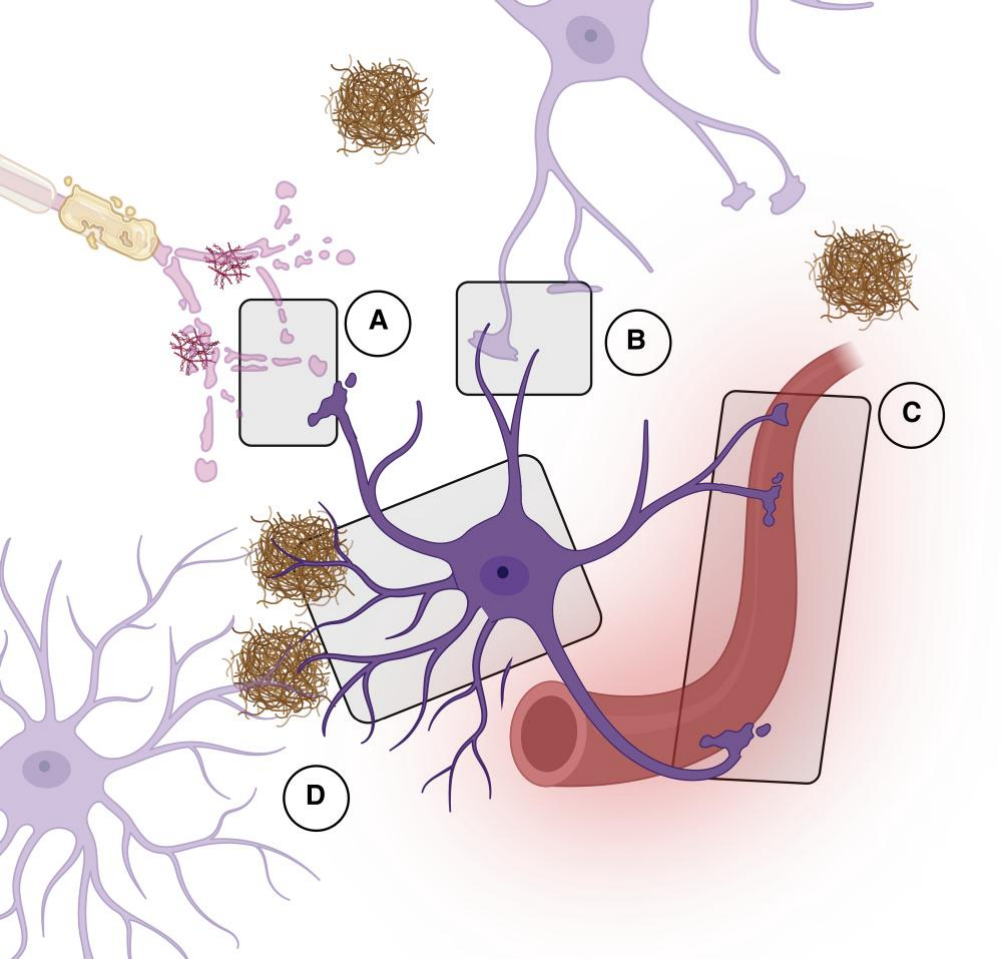
**Astrocytes roles and modifications in Alzheimer’s disease**. Due to their multifaced functions and implications in various brain structures, reactive astrocyte modifications deeply impact brain physiology at several levels. (**A**) *Tripartie synapses*: Alteration of neurotransmitter and glutamate pathways, modification of water and ion channel transport and release of inflammatory substances leading to synaptic disfunctions, neurotoxicity and neuroinflammation; (**B**) *Astrocytes tight junction*: Irregular ion transient (Ca^2+^, K^+^) and decreased gap junctions coupling; (**C**) *Astrocytes endfeet surrounding the blood–brain barrier (BBB)*: Release of overexpressed GFAP due to astrocytes impairment associated with BBB disruption and immune-cell massive infiltration; (**D**) *Astrocytes morphology*: Astrocytes structural atrophy and proliferation confining amyloid lesions and inducing Aβ clearance through the glymphatic pathway. Neurofibrillary tangles internalization.

These findings highlight a significant association between reactive astrogliosis and Alzheimer’s disease in which all brain cells are involved and interact in a deleterious synergetic manner that is still not fully understood. Consequently, astrocytes emerge as a highly promising target for the development of innovative therapeutic candidates and form a potential source of biomarkers for diagnosis and prognosis. However, astrogliosis represents only one aspect of the complex and dynamic neurodegenerative process. The multifaceted role of astrocytes in neurodegeneration is illustrated in Fig. 4.

### Relevance of GFAP as a clinical biomarker for Alzheimer’s disease

#### GFAP leakage in the bloodstream

GFAP is primarily localized intracellularly, but various mechanisms can induce its release into the extracellular space and subsequent entry to the bloodstream. The mechanisms underlying GFAP leakage into the blood under neurodegenerative conditions are complex and are not yet fully understood. Astrocytic damage or death due to astrogliosis and neuroinflammation is a prominent mechanism that may release GFAP into biofluids.^127,128^ Astrocyte endfeet, which align along brain capillaries and the BBB, may facilitate GFAP release into the bloodstream, particularly when the BBB is compromised.^129^ Disruption of the BBB, which occurs naturally with aging and is observed in early Alzheimer’s disease before the onset of dementia or neurodegeneration, could lead to increased levels of circulating GFAP and other soluble brain proteins.^130^ Multiple factors contribute to BBB disruption in Alzheimer’s disease, including neuroinflammation, the presence of APOE4 allele and amyloid deposition.^43^ The elevated risk of Alzheimer’s disease associated with APOE4 is partly due to its role in enhancing the permeability of brain vessels. Conversely, APOE2 is considered protective, maintaining BBB integrity.^131^ Dysregulation of calcium signalling and the release of inflammatory markers associated with neuroinflammation can also compromise BBB function by inducing the loss of tight junctions.^132^ In addition to its effects on the BBB, the APOE4 allele is strongly associated with cerebral amyloid angiopathy, a condition characterized by amyloid deposition along brain vessel walls. This condition is commonly observed in Alzheimer’s disease and contributes to the vascular pathology that exacerbates neurodegeneration. Increased secretion of Aβ_1–42_ and Aβ_1–40_ as well as reduced clearance due to glymphatic system impairment, induces aggregation within the parenchyma and along brain vasculature. This aggregation leads to BBB leakage, vessel occlusion or rupture, collectively worsening Alzheimer’s disease symptoms and cerebral impairment.^133^ The glymphatic system, a structured network involving astrocytes, may also provide a route for GFAP to enter the circulation.^134^ This system is essential for clearing the brain of metabolic waste and potentially harmful substances. Dysfunction of the glymphatic system in neurodegenerative diseases hinders the removal of pathogenic proteins like Aβ, leading to further aggregation.^135^ This disruption exacerbates neuroinflammation and oxidative stress, thereby driving disease progression.^136,137^ Recent evidence suggests that reactive astrocytes may also release GFAP through exocytosis, secreting small vesicles into extracellular space. These vesicles, capable of crossing the epithelial barrier of blood vessels, could contribute to the presence of circulating GFAP.^138^

#### GFAP as a clinical biomarker

Although pTau and Aβ are the most commonly used blood biomarkers for assessing amyloid pathologies and tauopathies in Alzheimer’s disease, recent studies have identified GFAP as a reliable alternative and potentially more sensitive indicator of pathological states. The implementation of next-generation immunoassays over the last decade has facilitated the rapid and reliable measurement of protein biomarkers in blood samples, enhancing the utility of CNS-derived markers. Given the pivotal role of astrogliosis in the pathogenesis of Alzheimer’s disease, growing evidence from large patient cohorts suggests that GFAP is a reliable biomarker for distinguishing Alzheimer’s disease from other neurodegenerative disorders even at early stages.^9,19^ Furthermore, GFAP is highly brain-specific and is not extensively secreted into biofluids under physiological conditions, reinforcing its relevance as a neurodegenerative brain disease biomarker.^139^

##### Blood GFAP levels differentiate Alzheimer’s disease from other neurodegenerative dementias

In 2013, within a moderate-size study including three 331 patients with different neurological conditions including brain concussion, infection, cancer or various dementias, Mayer *et al.*^140^ found that GFAP was reliably useful only for diagnosing intracranial haemorrhage and extensive intracranial bleeding. In other disease groups and controls, GFAP levels were close to the detection limit. Nevertheless, this study used a prototype Elecsys immunoassay (Roche) with a limit of quantification (0.05 µg/mL) considerably higher than the lower limit of quantification achieved with current technologies, such as single-molecule array (Simoa) platform (0.70 pg/mL, Quanterix), which might explain the lack of significant differences in GFAP levels for other pathologies. In 2018, the Brain Trauma Indicator, an assay measuring GFAP together with ubiquitin terminal-hydrolase-L1 in blood received Food and Drug Administration approval for assessing traumatic brain injury severity aiding the decision-making for CT scan use.^141^ With technical advances and the advent of fourth-generation immunoassays over the past decade, increasing evidence supports the diagnostic utility of plasma GFAP in distinguishing Alzheimer’s disease from other dementias. Despite reactive astrogliosis being a common pathological hallmark in both frontotemporal dementia and Alzheimer’s disease, significant differences were observed regarding blood GFAP level suggest that astrogliosis may differ between these pathologies. Oeckl *et al.*^21^ used Quanterix Simoa GFAP Discovery kit to measure serum GFAP and observed a 2-fold increase in Alzheimer’s disease (*n* = 230) group compared with the behavioural frontotemporal dementia group (*n* = 140, *P* < 0.001) and controls (*n* = 129, *P* < 0.001). Serum GFAP was also increased between early-stage mild cognitive impairment (MCI) individuals (*n* = 111) compared with those with behavioural frontotemporal dementia (*P* < 0.01) and controls (*P* < 0.001). This correlation was consistent across four sub-cohorts (Coimbra, Munich, Ulm and Nijmegen). Serum GFAP differentiated Alzheimer’s disease patients from control with an area under the curve (AUC) of 0.87 and from behavioural frontotemporal dementia with an AUC of 0.81, highlighting GFAP’s potential as a differential biomarker. Further studies have demonstrated the utility of plasma GFAP in differentiating other neurodegenerative dementias such as supranuclear palsy. Baiardi *et al.*^142^ found that plasma GFAP, measured using the Simoa SR-X platform, could differentiate Alzheimer’s disease from frontotemporal dementia and supranuclear palsy with respective receiver operating characteristic (ROC) curve AUCs of 0.82 and 0.77, respectively. However, differentiation between Alzheimer’s disease and corticobasal syndrome (AUC 0.62) or dementia with Lewy bodies (AUC 0.58) was less effective. Overall in this study, plasma GFAP showed strong performances, with AUCs ranging from 0.74 to 0.94, with the most consistent increases observed in Alzheimer’s disease.

##### Blood GFAP surpasses CSF GFAP

Unlike other common Alzheimer’s disease biomarkers, which typically perform better in CSF, plasma GFAP outperforms CSF GFAP in distinguishing between Aβ-positive and Aβ-negative individuals.^143,144^ Benedet *et al.*^129^ analysed three major cohorts: TRIAD (Canada, *n* = 300), ALFA + (Spain, *n* = 384) and Paris Labroisière BioCogBank (France, *n* = 187) using Simoa HD-X Single-Plex Assay (Quanterix). Their results showed that plasma GFAP provided greater accuracy than CSF GFAP in differentiating Aβ-positive from Aβ-negative individuals, with ROC curve AUCs ranging from 0.69 to 0.86 for plasma and 0.59 to 0.76 for CSF.^19^ This outcome is unexpected, as blood biomarkers for neurological conditions have traditionally been substitutes for CSF biomarkers. However, as discussed, the superior performance of plasma GFAP may reflect its more direct association with astrocytic activity and neurodegeneration, which might not be as readily captured through CSF measurements.

##### Blood GFAP correlates with amyloid build-up but not with tau pathology

Multiple studies suggest that plasma GFAP may be a biomarker for Aβ pathology (A+) rather than tau pathology (T+). In the ALFA+ cohort by Benedet *et al.,*^19^ plasma GFAP enabled the separation of A+T− individuals from A-T− individuals (*P* < 0.001). However, plasma GFAP concentrations in A-T+ individuals were not significantly higher compared with the A-T− group, suggesting that plasma GFAP levels are more closely associated with astrogliosis and amyloid build-up than with tau pathology. Pereira *et al.*^9^ corroborated these findings using the Swedish BioFINDER-2 cohort (*n* = 504) with the same assay. Elevated plasma GFAP levels correlated with Aβ-PET outcomes when tau-PET was included as a covariate across cognitively impaired individuals (*P* < 0.001), Aβ-positive cognitively unimpaired individuals (*P* = 0.007) and Aβ-positive cognitively impaired individuals (*P* = 0.041). Conversely, no significant correlation was observed between GFAP levels and tau-PET when Aβ-PET was included as a covariate. These findings underscore a specific association between amyloid pathology and elevated GFAP levels, independent of tau pathology. GFAP expression appears to be primarily associated with Aβ plaques, as supported by existing literature.^145^ Additionally, Rasing *et al.*^146^ recently demonstrated that elevated circulating GFAP levels strongly correlate with early cerebral amyloid angiopathy. These findings reinforce the hypothesis that GFAP leakage is more closely associated with amyloid pathology in brain and vasculature than with tau pathology.

##### Blood GFAP level correlates with cognitive impairment severity

In addition to its correlation with amyloid pathology, plasma GFAP levels also gradually increase with the severity of cognitive impairment across the Alzheimer’s disease continuum, as reported by Chatterjee *et al.*^20^ Their observations were based on the Australian Imaging, Biomarker and Lifestyle longitudinal cohort (AIBL, Australia) which included 181 individuals, with GFAP being quantified using the Quanterix Simoa Neurology-4-Plex Assay. Plasma GFAP was significantly higher in cognitively unimpaired Aβ-positive (CU- Aβ+), mild cognitively impaired Aβ-positive (MCI Aβ+) and Alzheimer’s disease Aβ-positive (AD Aβ+) groups (*P* < 0.0001) compared with cognitively unimpaired Aβ-negative (CU Aβ−) and mild cognitively impaired Aβ-negative groups (MCI Aβ−) (*P* < 0.0005). This suggests that elevated blood GFAP levels are associated with amyloid burden in Alzheimer’s disease, consistent with findings from other studies.^9,19^ Furthermore, GFAP levels also correlated with brain amyloid loading and disease severity, with significantly higher plasma GFAP in AD-Aβ+ compared with MCI Aβ+ (*P* < 0.001) and CU Aβ+ (*P* < 0.01) as well as between MCI Aβ+ against AD-Aβ+ (*P* < 0.001). Over the course of a 36-month longitudinal analysis within the same cohort, GFAP levels continued to rise in MCI Aβ+ and AD-Aβ+ compared with controls, further supporting that plasma GFAP levels track with disease progression. This study was the first to demonstrate increased plasma GFAP levels in cognitively normal older adults at risk of Alzheimer’s disease. Shen *et al.*^22^ further analysed a cross-sectional cohort of patients recruited from the Memory Clinic of the Huashan Hospital of Fudan University and the Chinese Alzheimer Biomarker and LifestylE (CABLE) study (China, *n* = 700) using Quanterix single-molecule array single plex assay. Their findings as aligned with previous cohort studies, showing that plasma GFAP was incrementally increased along the course of Alzheimer’s disease from preclinical and prodromal stages to Alzheimer’s disease dementia (*P* < 0.001).

##### Blood GFAP is elevated in at-risk cognitively unimpaired individuals

Chatterjee *et al.*^147^ had previously studied GFAP as a predictive biomarker for Alzheimer’s disease in at-risk individuals without cognitive impairment. In this study, plasma GFAP levels were compared between cognitively normal older adults at risk of Alzheimer’s disease with high PET-Aβ load (Aβ+) and those with low Aβ load (Aβ−) from the Kerr Anglican Retirement Village Initiative in Aging Health (KARVIAH) cohort (*n* = 134). Aβ+ participants demonstrated significantly higher plasma GFAP concentrations compared with Aβ−, both before and after adjusting for known risk factors such as age, sex and APOE ε_4_ status (*P* < 0.0001). These results suggest that elevated plasma GFAP levels could serve as an early indicator for predicting Alzheimer’s disease development in at-risk individuals before the onset of cognitive decline. Through a longitudinal analysis within a cohort from the Shanghai Aging Study (*n* = 118), Shen *et al.*^22^ showed that cognitively normal individuals who later developed Alzheimer’s disease presented higher baseline GFAP compared with those who did not convert to Alzheimer’s disease. The difference between converters and non-converters was significant (*P* < 0.001), with an AUC of 0.85. These findings support the utility of plasma GFAP as a predictive biomarker for Alzheimer’s disease, even before the onset of cognitive decline.

### Diagnosis perspectives of GFAP as a blood-based biomarker

#### Blood biomarkers: what they offer and linked challenges

CSF has historically been the preferred sample type for neurodegenerative disease research due to its proximity to the brain. However, its collection via lumbar puncture is invasive, and the volume that can be collected is limited. Imaging techniques such as PET scan, while effective, are costly, have low throughput and involve irradiation which reduces its large-scale implementation. Recently, significant efforts have been made to quantify brain-derived proteins in blood, offering a less invasive alternative. Blood sampling is advantageous due to lower costs and higher throughput. Nevertheless, the concentrations of brain-derived proteins in blood are extremely low compared with abundant blood proteins such as albumin and immunoglobulins. Consequently, blood-based biomarker testing requires highly sensitive measurement devices and meticulous pre-analytical handling to achieve the necessary performance standards.^148^

In clinical practice, blood biomarkers hold promise for the diagnosis, prognosis and monitoring of Alzheimer’s disease. These biomarkers offer several advantages, including reduced invasiveness, ease of sample collection, affordability, rapid implementation and broad acceptance. The accessibility of blood samples could also facilitate research into potential treatments, evaluation of therapeutic efficacy and a deeper understanding of the disease’s pathological landscape, thereby serving as a promising strategy for screening. Despite the high accuracy of current clinically approved diagnosis and screening methods, such as amyloid and tau-PET imaging and CSF protein quantification, their limited availability, high cost and invasiveness urge the need for surrogate biomarkers for screening and diagnosis purposes.

#### Current and future directions: the ATNIVS framework

**Figure 5:**
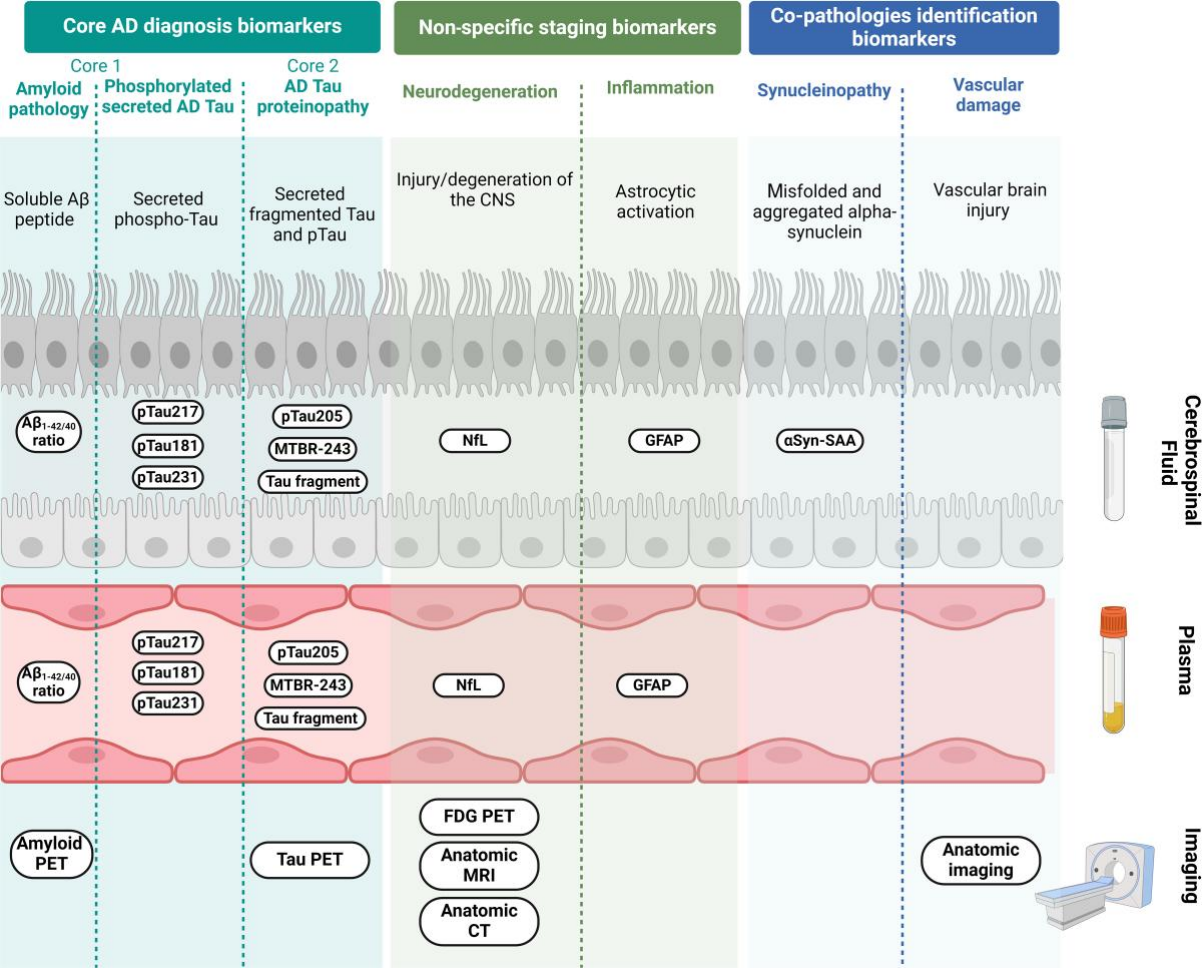
**ATNISV classification and current diagnosis tools for Alzheimer’s disease**. The ATNIVS Alzheimer’s disease (AD) diagnosis framework draft suggested in 2023 amend former ATN classification described by NIA-AA. It includes four categories to cover a broader spectrum of the pathophysiological complexity of Alzheimer’s disease and relevant biological and imaging related biomarkers. The Core 1 (‘A’) and Core 2 (‘T’) biomarkers categories reflects Alzheimer’s disease main hallmarks: amyloid build-up and neurofibrillary tangles. The measurement in biofluids of Aβ_1–42/40_ ratio, secreted phosphorylated tau at positions 181, 217, 231 and 205 as well as tau fragments and specific tau regions such as microtubule binding region-243 (MTBR-243), respectively, characterize amyloid and tau pathology. Within this core AD biomarkers, amyloid and tau-PET imaging are used to visualize aggregates location and spreading. Other divisions includes neurodegeneration (‘N’) and neuroinflammation (‘I’) both reflecting non-specific hallmarks related to disease stage. The ‘N’ category stands for neurodegeneration which can be evaluated using CSF or plasma neurofilament light chain (NfL) protein along with anatomic (MRI, CT) or metabolism [^18^F]-fluorodeoxyglucose (FDG)-PET imaging. The ‘I’ section refers to neuroinflammation stated through glial fibrillary acid protein measurement in biofluids and specifically associated with astrogliosis. The two last additional categories were thought to map potential coexistent co-pathologies adding to the neurodegenerative burden. Hence, ‘S’ category characterize synucleinopathies entwined with aggregated abnormal alpha-synuclein in CSF evidenced with alpha-synuclein seed amplification assay (αsyn-SAA) and ‘V’ refers to vascular damages which can be examined using anatomic imaging.

In accordance with The National Institute of Aging and Alzheimer’s Association (NIA-AA) framework Alzheimer’s disease is a biological construct that does not solely rely on clinical symptoms. Diagnosis involves identifying core pathological hallmarks and related biomarkers. The initial guidelines, published in 2011, have recently been updated.^10^ According to these guidelines, Alzheimer’s disease biomarkers are categorized using the [AT(*N*)] classification system. ‘A’ refers to Aβ peptide deposition, identified through low levels of Aβ_1–42_, a decreased Aβ_1–42/40_ ratio and positive amyloid PET. ‘T’ represents the accumulation of abnormal pathologic tau protein in the brain, which is reflected in increased CSF concentration of phosphorylated tau and positive tau-PET. ‘N’ encompasses neurodegeneration markers, including brain atrophy detected via magnetic resonance imaging (MRI), [^18^F]-fluorodeoxyglucose (FDG)-PET and CSF total-tau level. The ‘N’ category continues to evolve with the potential addition of biomarkers linked to reactive astrogliosis.^11^ However, this framework has not fully captured the complexity of Alzheimer’s disease. To address this, a new category ‘X’ was proposed in 2021. The ‘X’ category aims to integrate novel candidate biomarkers reflecting additional pathological mechanisms, such as neuroimmune imbalance, synaptic dysfunctions and BBB alterations.^5^ At the Alzheimer’s Association International Conference (AAIC) 2023 in Amsterdam, Jack Clifford of the Rochester Clinic Minnesota presented the draft revision of the Alzheimer’s disease diagnostic criteria, updating the 2018 research framework to incorporate recent advancements.^10^ The revised framework, dubbed ATNIVS, retains A and T were retained as core diagnosis markers while *N* has been relegated to a second-tier marker due to its non-specificity. The framework introduces ‘I’ category characterizing inflammation as well as two additional categories associated with common non-Alzheimer’s disease pathologies: ‘S’ for synucleinopathies and ‘V’ for vascular brain injury. Furthermore, this framework incorporates blood biomarkers alongside with existing CSF and PET imaging biomarkers.^23^ The full ATNIVS framework is presented in Fig. 5. It is increasingly recognized that Alzheimer’s disease represents a spectrum of disorders with various genetic and potentially environmental risk factors that share similar and interrelated pathological and clinical features.^149^

#### Potential application of GFAP in Alzheimer’s disease diagnosis and disease monitoring

Plasma GFAP is increasingly recognized as a valuable biomarker that can be integrated into the existing diagnostic framework for Alzheimer’s disease. While GFAP alone reflects astrocytic activation and neuroinflammation, which are common across various neurodegenerative diseases, its diagnostic utility is significantly enhanced when used alongside with established Alzheimer’s disease biomarkers. Studies support the combined use of GFAP with other blood-based biomarkers to improve diagnostic accuracy, particularly in cases involving overlapping comorbidities, progressive neurodegeneration and amyloid accumulation.^150^ Therefore biomarkers like NfL,^151^ associated with general neuronal damage, and Alzheimer’s disease-specific biomarkers such as the Aβ_1–42/40_ ratio and pTau-181 have been explored in conjunction with GFAP.^152,153^ In a cohort with 252 subjects from Amsterdam Dementia Cohort, Verberk *et al.*^154^ demonstrated that combining multiple biomarkers enhances diagnostic accuracy. Using the Simoa 4-Plex assay (Quanterix), they found that the ROC curve AUCs for predicting amyloid PET status were 0.71 for NfL, 0.81 for GFAP and 0.73 for the Aβ_1–42_/Aβ_1–40_ ratio. When these markers were combined, the AUC increased to 0.88. Similarly, Yang *et al.*^155^ observed that combining pTau-181 with GFAP provided superior results for distinguishing amyloid status. With individual AUCs of 0.81 and 0.82, respectively, and a combined AUC of 0.86 in the Nevada Center for Neurodegeneration and Translational Neuroscience cohort (United States, *n* = 144). Among cognitively impaired participants (*n* = 87) the AUC further to 0.93, only marginal enhancement (0.01) upon addition of NfL. For differentiating frontotemporal dementia from cognitively unimpaired or non-neurodegenerative impairment, plasma NfL was quite accurate as corroborated by Sarto *et al.*^156^ in a study involving 385 individuals from Alzheimer’s disease and other cognitive disorders unit (Barcelona), with AUCs of 0.90 and 0.93. In this study, GFAP showed similar performances to pTau-181 in distinguishing Alzheimer’s disease from CU (*P* < 0.001) and MCI (*P* < 0.001) individuals, with a strong correlation with Aβ status. Additionally, GFAP both effectively distinguished Alzheimer’s disease from other neurodegenerative dementias, such as dementia with Lewy bodies (*P* < 0.001). In contrast, total-tau did not significantly differentiate between these pathologies. Integrating GFAP into the existing ATN framework could enhance its ability to reflect the complex pathology of Alzheimer’s disease, providing valuable insights into the role of reactive astrogliosis in Alzheimer’s disease. Furthermore, apart from brain-related proteins, including additional biomarkers, into these analyses could improve patient care by addressing comorbid conditions like kidney dysfunction. This is particularly important in elderly patients who might suffer from other chronic conditions that could impair Alzheimer’s disease diagnosis.^157^

## Conclusion

Astrogliosis has emerged as a significant feature of Alzheimer’s disease, characterized by the proliferation of structurally and functionally altered astrocytes. This pathological process is associated with the overexpression and release of GFAP in biofluids, which correlates with disease severity and progression. Plasma GFAP has demonstrated superior performance compared with its CSF counterpart in diagnosing Alzheimer’s disease, particularly in distinguishing Aβ-positive from Aβ-negative individuals, providing prognostic information in at-risk populations and differentiating Alzheimer’s disease from other neurodegenerative disorders such as frontotemporal dementia. These findings underscore the clinical relevance of GFAP as a biomarker for Alzheimer’s disease-related astrogliosis.

Although the potential of targeting reactive astrocytes as a therapeutic strategy remains underexplored, they may represent promising candidates for therapies aimed at halting disease progression due to their early and prominent role in Alzheimer’s pathology. However, while GFAP is a valuable biomarker, it is insufficient as a standalone marker in clinical practice. Incorporating GFAP into a panel of biomarkers enhances diagnostic accuracy for Alzheimer’s disease by providing additional insights into astrocytic activation and neuroinflammation, which are critical aspects of the disease’s pathology. This approach not only aids in early diagnosis and monitoring of disease progression but also helps differentiate Alzheimer’s disease from other neurodegenerative conditions. By integrating GFAP within a multi-biomarker strategy, clinical practice can advance towards more precise and personalized management of Alzheimer’s disease.

## Data Availability

Data sharing is not applicable to this article as no new data were created or analysed in this study.

## References

[1] Nichols E, Steinmetz JD, Vollset SE, et al Estimation of the global prevalence of dementia in 2019 and forecasted prevalence in 2050: An analysis for the Global Burden of Disease Study 2019. Lancet Public Health. 2022;7(2):e105–e125. 34998485 10.1016/S2468-2667(21)00249-8 PMC8810394

[2] Serrano-Pozo A, Frosch MP, Masliah E, Hyman BT. Neuropathological alterations in Alzheimer disease. Cold Spring Harb Perspect Med. 2011;1(1):a006189. 22229116 10.1101/cshperspect.a006189 PMC3234452

[3] Yiannopoulou KG, Anastasiou AI, Zachariou V, Pelidou SH. Reasons for failed trials of disease-modifying treatments for Alzheimer disease and their contribution in recent research. Biomedicines. 2019;7(4):97. 31835422 10.3390/biomedicines7040097 PMC6966425

[4] Kim CK, Lee YR, Ong L, Gold M, Kalali A, Sarkar J. Alzheimer’s disease: Key insights from two decades of clinical trial failures. J Alzheimers Dis. 2022;87(1):83–100. 35342092 10.3233/JAD-215699 PMC9198803

[5] Hampel H, Hu Y, Cummings J, et al Blood-based biomarkers for Alzheimer’s disease: Current state and future use in a transformed global healthcare landscape. Neuron. 2023;111(18):2781–2799. 37295421 10.1016/j.neuron.2023.05.017 PMC10720399

[6] Brand AL, Lawler PE, Bollinger JG, et al The performance of plasma amyloid beta measurements in identifying amyloid plaques in Alzheimer’s disease: A literature review. Alzheimers Res Ther. 2022;14(1):195. 36575454 10.1186/s13195-022-01117-1 PMC9793600

[7] Barthélemy NR, Horie K, Sato C, Bateman RJ. Blood plasma phosphorylated-tau isoforms track CNS change in Alzheimer’s disease. J Exp Med. 2020;217(11):e20200861. 32725127 10.1084/jem.20200861 PMC7596823

[8] Delaby C, Bousiges O, Bouvier D, et al Neurofilaments: A key new biomarker for clinicians. Part 2: Neurofilaments, an asset beyond neurodegenerative diseases. Ann Biol Clin (Paris). 2022;80(5):441–450. 36453743 10.1684/abc.2022.1758

[9] Pereira JB, Janelidze S, Smith R, et al Plasma GFAP is an early marker of amyloid-β but not tau pathology in Alzheimer’s disease. Brain. 2021;144(11):3505–3516. 34259835 10.1093/brain/awab223 PMC8677538

[10] Jack CR, Bennett DA, Blennow K, et al NIA-AA Research framework: Toward a biological definition of Alzheimer’s disease. Alzheimers Dement. 2018;14(4):535–562. 29653606 10.1016/j.jalz.2018.02.018 PMC5958625

[11] Jain P, Wadhwa PK, Jadhav HR. Reactive astrogliosis: Role in Alzheimer’s disease. CNS Neurol Disord Drug Targets. 2015;14(7):872–879. 26166438 10.2174/1871527314666150713104738

[12] Sofroniew MV . Astrogliosis. Cold Spring Harb Perspect Biol. 2014;7(2):a020420. 25380660 10.1101/cshperspect.a020420 PMC4315924

[13] Abdelhak A, Foschi M, Abu-Rumeileh S, et al Blood GFAP as an emerging biomarker in brain and spinal cord disorders. Nat Rev Neurol. 2022;18(3):158–172. 35115728 10.1038/s41582-021-00616-3

[14] Palombo F, Tamagnini F, Jeynes JCG, et al Detection of Aβ plaque-associated astrogliosis in Alzheimer’s disease brain by spectroscopic imaging and immunohistochemistry. Analyst. 2018;143(4):850–857. 29230441 10.1039/c7an01747b PMC5851084

[15] Frost GR, Li YM. The role of astrocytes in amyloid production and Alzheimer’s disease. Open Biol. 2017;7(12):170228. 29237809 10.1098/rsob.170228 PMC5746550

[16] Carter SF, Schöll M, Almkvist O, et al Evidence for astrocytosis in prodromal Alzheimer disease provided by ^11^C-deuterium-L-deprenyl: A multitracer PET paradigm combining ^11^C-Pittsburgh compound B and ^18^F-FDG. J Nucl Med. 2012;53(1):37–46. 22213821 10.2967/jnumed.110.087031

[17] Rodriguez-Vieitez E, Ni R, Gulyás B, et al Astrocytosis precedes amyloid plaque deposition in Alzheimer APPswe transgenic mouse brain: A correlative positron emission tomography and in vitro imaging study. Eur J Nucl Med Mol Imaging. 2015;42(7):1119–1132. 25893384 10.1007/s00259-015-3047-0 PMC4424277

[18] Schöll M, Carter SF, Westman E, et al Early astrocytosis in autosomal dominant Alzheimer’s disease measured in vivo by multi-tracer positron emission tomography. Sci Rep. 2015;5:16404. 26553227 10.1038/srep16404 PMC4639762

[19] Benedet AL, Milà-Alomà M, Vrillon A, et al Differences between plasma and cerebrospinal fluid glial fibrillary acidic protein levels across the Alzheimer disease continuum. JAMA Neurol. 2021;78(12):1471–1483. 34661615 10.1001/jamaneurol.2021.3671 PMC8524356

[20] Chatterjee P, Vermunt L, Gordon BA, et al Plasma glial fibrillary acidic protein in autosomal dominant Alzheimer’s disease: Associations with Aβ-PET, neurodegeneration, and cognition. Alzheimers Dement. 2023;19(7):2790–2804. 36576155 10.1002/alz.12879 PMC10300233

[21] Oeckl P, Halbgebauer S, Anderl-Straub S, et al Glial fibrillary acidic protein in serum is increased in Alzheimer’s disease and correlates with cognitive impairment. J Alzheimers Dis. 2019;67(2):481–488. 30594925 10.3233/JAD-180325

[22] Shen XN, Huang SY, Cui M, et al Plasma glial fibrillary acidic protein in the Alzheimer disease continuum: Relationship to other biomarkers, differential diagnosis, and prediction of clinical progression. Clin Chem. 2023;69(4):411–421. 36861369 10.1093/clinchem/hvad018

[23] Revised Again: Alzheimer’s Diagnostic Criteria Get Another Makeover | ALZFORUM. Accessed 12 December 2023. https://www.alzforum.org/news/conference-coverage/revised-again-alzheimers-diagnostic-criteria-get-another-makeover#top

[24] Eng LF, Vanderhaeghen JJ, Bignami A, Gerstl B. An acidic protein isolated from fibrous astrocytes. Brain Res. 1971;28(2):351–354. 5113526 10.1016/0006-8993(71)90668-8

[25] Abbott NJ, Rönnbäck L, Hansson E. Astrocyte–endothelial interactions at the blood–brain barrier. Nat Rev Neurosci. 2006;7(1):41–53. 16371949 10.1038/nrn1824

[26] Attwell D, Buchan AM, Charpak S, Lauritzen M, Macvicar BA, Newman EA. Glial and neuronal control of brain blood flow. Nature. 2010;468(7321):232–243. 21068832 10.1038/nature09613 PMC3206737

[27] Beard E, Lengacher S, Dias S, Magistretti PJ, Finsterwald C. Astrocytes as key regulators of brain energy metabolism: New therapeutic perspectives. Front Physiol. 2022;12:825816, Accessed 23 November 2023. https://www.frontiersin.org/articles/10.3389/fphys.2021.825816. 35087428 10.3389/fphys.2021.825816 PMC8787066

[28] Wang S, Wang B, Shang D, Zhang K, Yan X, Zhang X. Ion channel dysfunction in astrocytes in neurodegenerative diseases. Front Physiol. 2022;13:814285. 35222082 10.3389/fphys.2022.814285 PMC8864228

[29] Mahmoud S, Gharagozloo M, Simard C, Gris D. Astrocytes maintain glutamate homeostasis in the CNS by controlling the balance between glutamate uptake and release. Cells. 2019;8(2):184. 30791579 10.3390/cells8020184 PMC6406900

[30] Parpura V, Basarsky TA, Liu F, Jeftinija K, Jeftinija S, Haydon PG. Glutamate-mediated astrocyte-neuron signalling. Nature. 1994;369(6483):744–747. 7911978 10.1038/369744a0

[31] Sofroniew MV, Vinters HV. Astrocytes: Biology and pathology. Acta Neuropathol. 2010;119(1):7–35. 20012068 10.1007/s00401-009-0619-8 PMC2799634

[32] Mohaupt P, Vialaret J, Hirtz C, Lehmann S. Readthrough isoform of aquaporin-4 (AQP4) as a therapeutic target for Alzheimer’s disease and other proteinopathies. Alzheimers Res Ther. 2023;15(1):170. 37821965 10.1186/s13195-023-01318-2 PMC10566184

[33] Tarasoff-Conway JM, Carare RO, Osorio RS, et al Clearance systems in the brain—Implications for Alzheimer disease. Nat Rev Neurol. 2015;11(8):457–470. 26195256 10.1038/nrneurol.2015.119 PMC4694579

[34] Sapkota D, Florian C, Doherty BM, et al Aqp4 stop codon readthrough facilitates amyloid-β clearance from the brain. Brain. 2022;145(9):2982–2990. 36001414 10.1093/brain/awac199 PMC10233234

[35] Nägler K, Mauch DH, Pfrieger FW. Glia-derived signals induce synapse formation in neurones of the rat central nervous system. J Physiol. 2001;533(Pt 3):665–679. 11410625 10.1111/j.1469-7793.2001.00665.x PMC2278670

[36] Barres BA . The mystery and magic of glia: A perspective on their roles in health and disease. Neuron. 2008;60(3):430–440. 18995817 10.1016/j.neuron.2008.10.013

[37] Tabata H . Diverse subtypes of astrocytes and their development during corticogenesis. Front Neurosci. 2015;9:114. 25904839 10.3389/fnins.2015.00114 PMC4387540

[38] Köhler S, Winkler U, Hirrlinger J. Heterogeneity of astrocytes in grey and white matter. Neurochem Res. 2021;46(1):3–14. 31797158 10.1007/s11064-019-02926-x

[39] Hawrylycz MJ, Lein ES, Guillozet-Bongaarts AL, et al An anatomically comprehensive atlas of the adult human brain transcriptome. Nature. 2012;489(7416):391–399. 22996553 10.1038/nature11405 PMC4243026

[40] Hasel P, Rose IVL, Sadick JS, Kim RD, Liddelow SA. Neuroinflammatory astrocyte subtypes in the mouse brain. Nat Neurosci. 2021;24(10):1475–1487. 34413515 10.1038/s41593-021-00905-6

[41] Hasel P, Cooper ML, Marchildon AE, et al Defining the molecular identity and morphology of glia limitans superficialis astrocytes in mouse and human. bioRxiv 535893. doi:10.1101/2023.04.06.535893, 6 April 2023, preprint: not peer reviewed. PMC12160017 39982817

[42] Wu YE, Pan L, Zuo Y, Li X, Hong W. Detecting activated cell populations using single-cell RNA-Seq. Neuron. 2017;96(2):313–329.e6. 29024657 10.1016/j.neuron.2017.09.026

[43] Obermeier B, Daneman R, Ransohoff RM. Development, maintenance and disruption of the blood-brain barrier. Nat Med. 2013;19(12):1584–1596. 24309662 10.1038/nm.3407 PMC4080800

[44] Quintana FJ . Astrocytes to the rescue! Glia limitans astrocytic endfeet control CNS inflammation. J Clin Invest. 2017;127(8):2897–2899. 28737511 10.1172/JCI95769 PMC5531401

[45] Batiuk MY, Martirosyan A, Wahis J, et al Identification of region-specific astrocyte subtypes at single cell resolution. Nat Commun. 2020;11(1):1220. 32139688 10.1038/s41467-019-14198-8 PMC7058027

[46] Feinstein DL, Weinmaster GA, Milner RJ. Isolation of cDNA clones encoding rat glial fibrillary acidic protein: Expression in astrocytes and in Schwann cells. J Neurosci Res. 1992;32(1):1–14. 1629938 10.1002/jnr.490320102

[47] Reeves SA, Helman LJ, Allison A, Israel MA. Molecular cloning and primary structure of human glial fibrillary acidic protein. Proc Natl Acad Sci U S A. 1989;86(13):5178–5182. 2740350 10.1073/pnas.86.13.5178 PMC297581

[48] Kumanishi T, Usui H, Ichikawa T, et al Human glial fibrillary acidic protein (GFAP): Molecular cloning of the complete cDNA sequence and chromosomal localization (chromosome 17) of the GFAP gene. Acta Neuropathol. 1992;83(6):569–578. 1636374 10.1007/BF00299404

[49] van Asperen JV, Robe PAJT, Hol EM. GFAP alternative splicing and the relevance for disease – A focus on diffuse gliomas. ASN Neuro. 2022;14:17590914221102064. 10.1177/17590914221102065 PMC9185002 35673702

[50] Brenner M, Lampel K, Nakatani Y, et al Characterization of human cDNA and genomic clones for glial fibrillary acidic protein. Brain Res Mol Brain Res. 1990;7(4):277–286. 2163003 10.1016/0169-328x(90)90078-r

[51] Deka H, Sarmah R, Sharma A, Biswas S. Modelling and characterization of glial fibrillary acidic protein. Bioinformation. 2015;11(8):393–400. 26420920 10.6026/97320630011393 PMC4574122

[52] Roelofs RF, Fischer DF, Houtman SH, et al Adult human subventricular, subgranular, and subpial zones contain astrocytes with a specialized intermediate filament cytoskeleton. Glia. 2005;52(4):289–300. 16001427 10.1002/glia.20243

[53] Galea E, Dupouey P, Feinstein DL. Glial fibrillary acidic protein mRNA isotypes: Expression in vitro and in vivo. J Neurosci Res. 1995;41(4):452–461. 7473876 10.1002/jnr.490410404

[54] Riol H, Tardy M, Rolland B, Lévesque G, Murthy MR. Detection of the peripheral nervous system (PNS)-type glial fibrillary acidic protein (GFAP) and its mRNA in human lymphocytes. J Neurosci Res. 1997;48(1):53–62. 9086181

[55] Zelenika D, Grima B, Brenner M, Pessac B. A novel glial fibrillary acidic protein mRNA lacking exon 1. Brain Res Mol Brain Res. 1995;30(2):251–258. 7637576 10.1016/0169-328x(95)00010-p

[56] Condorelli DF, Nicoletti VG, Barresi V, et al Structural features of the rat GFAP gene and identification of a novel alternative transcript. J Neurosci Res. 1999;56(3):219–228. 10336251 10.1002/(SICI)1097-4547(19990501)56:3<219::AID-JNR1>3.0.CO;2-2

[57] Nielsen AL, Holm IE, Johansen M, Bonven B, Jørgensen P, Jørgensen AL. A new splice variant of glial fibrillary acidic protein, GFAP epsilon, interacts with the presenilin proteins. J Biol Chem. 2002;277(33):29983–29991. 12058025 10.1074/jbc.M112121200

[58] van Strien ME, Sluijs JA, Reynolds BA, Steindler DA, Aronica E, Hol EM. Isolation of neural progenitor cells from the human adult subventricular zone based on expression of the cell surface marker CD271. Stem Cells Transl Med. 2014;3(4):470–480. 24604282 10.5966/sctm.2013-0038 PMC3973708

[59] Van Den Berge SA, Middeldorp J, Zhang CE, et al Longterm quiescent cells in the aged human subventricular neurogenic system specifically express GFAP-δ. Aging Cell. 2010;9(3):313–326. 20121722 10.1111/j.1474-9726.2010.00556.x

[60] Kamphuis W, Mamber C, Moeton M, et al GFAP isoforms in adult mouse brain with a focus on neurogenic astrocytes and reactive astrogliosis in mouse models of Alzheimer disease. PLoS ONE. 2012;7(8):e42823. 22912745 10.1371/journal.pone.0042823 PMC3418292

[61] Blechingberg J, Holm IE, Nielsen KB, Jensen TH, Jørgensen AL, Nielsen AL. Identification and characterization of GFAPkappa, a novel glial fibrillary acidic protein isoform. Glia. 2007;55(5):497–507. 17203480 10.1002/glia.20475

[62] Messing A, Brenner M. GFAP at 50. ASN Neuro. 2020;12:1759091420949680. 32811163 10.1177/1759091420949680 PMC7440737

[63] Helman G, Takanohashi A, Hagemann TL, et al Type II Alexander disease caused by splicing errors and aberrant overexpression of an uncharacterized GFAP isoform. Hum Mutat. 2020;41(6):1131–1137. 32126152 10.1002/humu.24008 PMC7491703

[64] van Bodegraven EJ, Sluijs JA, Tan AK, Robe PAJT, Hol EM. New GFAP splice isoform (GFAPµ) differentially expressed in glioma translates into 21 kDa N-terminal GFAP protein. FASEB J. 2021;35(3):e21389. 33583081 10.1096/fj.202001767R PMC12266313

[65] Middeldorp J, Hol EM. GFAP in health and disease. Prog Neurobiol. 2011;93(3):421–443. 21219963 10.1016/j.pneurobio.2011.01.005

[66] Kamphuis W, Middeldorp J, Kooijman L, et al Glial fibrillary acidic protein isoform expression in plaque related astrogliosis in Alzheimer’s disease. Neurobiol Aging. 2014;35(3):492–510. 24269023 10.1016/j.neurobiolaging.2013.09.035

[67] Guzenko D, Chernyatina AA, Strelkov SV. Crystallographic studies of intermediate filament proteins. Subcell Biochem. 2017;82:151–170. 28101862 10.1007/978-3-319-49674-0_6

[68] Kim B, Kim S, Jin MS. Crystal structure of the human glial fibrillary acidic protein 1B domain. Biochem Biophys Res Commun. 2018;503(4):2899–2905. 30126635 10.1016/j.bbrc.2018.08.066

[69] Snider NT, Omary MB. Post-translational modifications of intermediate filament proteins: Mechanisms and functions. Nat Rev Mol Cell Biol. 2014;15(3):163–177. 24556839 10.1038/nrm3753 PMC4079540

[70] Inagaki M, Gonda Y, Nishizawa K, et al Phosphorylation sites linked to glial filament disassembly in vitro locate in a non-alpha-helical head domain. J Biol Chem. 1990;265(8):4722–4729. 2155236

[71] Battaglia RA, Beltran AS, Delic S, et al Site-specific phosphorylation and caspase cleavage of GFAP are new markers of Alexander disease severity. eLife. 2019;8:e47789. 31682229 10.7554/eLife.47789 PMC6927689

[72] Jin Z, Fu Z, Yang J, Troncosco J, Everett AD, Van Eyk JE. Identification and characterization of citrulline-modified brain proteins by combining HCD and CID fragmentation. Proteomics. 2013;13(17):2682–2691. 23828821 10.1002/pmic.201300064 PMC4864592

[73] Faigle W, Cruciani C, Wolski W, et al Brain citrullination patterns and T cell reactivity of cerebrospinal fluid-derived CD4^+^ T cells in multiple sclerosis. Front Immunol. 2019;10:540. 31024521 10.3389/fimmu.2019.00540 PMC6467957

[74] Viedma-Poyatos Á, de Pablo Y, Pekny M, Pérez-Sala D. The cysteine residue of glial fibrillary acidic protein is a critical target for lipoxidation and required for efficient network organization. Free Radic Biol Med. 2018;120:380–394. 29635011 10.1016/j.freeradbiomed.2018.04.007

[75] Bignami A, Eng LF, Dahl D, Uyeda CT. Localization of the glial fibrillary acidic protein in astrocytes by immunofluorescence. Brain Res. 1972;43(2):429–435. 4559710 10.1016/0006-8993(72)90398-8

[76] Etienne-Manneville S . Cytoplasmic intermediate filaments in cell biology. Annu Rev Cell Dev Biol. 2018;34(1):1–28. 30059630 10.1146/annurev-cellbio-100617-062534

[77] Hol EM, Capetanaki Y. Type III intermediate filaments desmin, glial fibrillary acidic protein (GFAP), vimentin, and peripherin. Cold Spring Harb Perspect Biol. 2017;9(12):a021642. 29196434 10.1101/cshperspect.a021642 PMC5710105

[78] Lowery J, Kuczmarski ER, Herrmann H, Goldman RD. Intermediate filaments play a pivotal role in regulating cell architecture and function. J Biol Chem. 2015;290(28):17145–17153. 25957409 10.1074/jbc.R115.640359 PMC4498054

[79] Gomi H, Yokoyama T, Fujimoto K, et al Mice devoid of the glial fibrillary acidic protein develop normally and are susceptible to scrapie prions. Neuron. 1995;14(1):29–41. 7826639 10.1016/0896-6273(95)90238-4

[80] Liedtke W, Edelmann W, Bieri PL, et al GFAP is necessary for the integrity of CNS white matter architecture and long-term maintenance of myelination. Neuron. 1996;17(4):607–615. 8893019 10.1016/s0896-6273(00)80194-4

[81] Nawashiro H, Messing A, Azzam N, Brenner M. Mice lacking GFAP are hypersensitive to traumatic cerebrospinal injury. Neuroreport. 1998;9(8):1691–1696. 9665584 10.1097/00001756-199806010-00004

[82] Bandyopadhyay U, Sridhar S, Kaushik S, Kiffin R, Cuervo AM. Identification of regulators of chaperone-mediated autophagy. Mol Cell. 2010;39(4):535–547. 20797626 10.1016/j.molcel.2010.08.004 PMC2945256

[83] Perng MD, Cairns L, van den IJssel P, Prescott A, Hutcheson AM, Quinlan RA. Intermediate filament interactions can be altered by HSP27 and alphaB-crystallin. J Cell Sci. 1999;112(Pt 13):2099–2112. 10362540 10.1242/jcs.112.13.2099

[84] Vinci L, Ravarino A, Fanos V, et al Immunohistochemical markers of neural progenitor cells in the early embryonic human cerebral cortex. Eur J Histochem. 2016;60(1):2563. 26972711 10.4081/ejh.2016.2563 PMC4800247

[85] Pekny M, Eliasson C, Siushansian R, et al The impact of genetic removal of GFAP and/or vimentin on glutamine levels and transport of glucose and ascorbate in astrocytes. Neurochem Res. 1999;24(11):1357–1362. 10555775 10.1023/a:1022572304626

[86] Eliasson C, Sahlgren C, Berthold CH, et al Intermediate filament protein partnership in astrocytes. J Biol Chem. 1999;274(34):23996–24006. 10446168 10.1074/jbc.274.34.23996

[87] Oberheim NA, Takano T, Han X, et al Uniquely hominid features of adult human astrocytes. J Neurosci. 2009;29(10):3276–3287. 19279265 10.1523/JNEUROSCI.4707-08.2009 PMC2819812

[88] Burda JE, Sofroniew MV. Reactive gliosis and the multicellular response to CNS damage and disease. Neuron. 2014;81(2):229–248. 24462092 10.1016/j.neuron.2013.12.034 PMC3984950

[89] Rostami J, Fotaki G, Sirois J, et al Astrocytes have the capacity to act as antigen-presenting cells in the Parkinson’s disease brain. J Neuroinflammation. 2020;17(1):119. 32299492 10.1186/s12974-020-01776-7 PMC7164247

[90] Baert L, Benkhoucha M, Popa N, et al A proliferation-inducing ligand–mediated anti-inflammatory response of astrocytes in multiple sclerosis. Ann Neurol. 2019;85(3):406–420. 30635946 10.1002/ana.25415

[91] Wang H, Song G, Chuang H, et al Portrait of glial scar in neurological diseases. Int J Immunopathol Pharmacol. 2018;31:2058738418801406. 30309271 10.1177/2058738418801406 PMC6187421

[92] Sofroniew MV . Astrocyte barriers to neurotoxic inflammation. Nat Rev Neurosci. 2015;16(5):249–263. 25891508 10.1038/nrn3898 PMC5253239

[93] Kawano H, Kimura-Kuroda J, Komuta Y, et al Role of the lesion scar in the response to damage and repair of the central nervous system. Cell Tissue Res. 2012;349(1):169–180. 22362507 10.1007/s00441-012-1336-5 PMC3375417

[94] Götz M, Hartfuss E, Malatesta P. Radial glial cells as neuronal precursors: A new perspective on the correlation of morphology and lineage restriction in the developing cerebral cortex of mice. Brain Res Bull. 2002;57(6):777–788. 12031274 10.1016/s0361-9230(01)00777-8

[95] Malatesta P, Hartfuss E, Götz M. Isolation of radial glial cells by fluorescent-activated cell sorting reveals a neuronal lineage. Development. 2000;127(24):5253–5263. 11076748 10.1242/dev.127.24.5253

[96] Aquino DA, Padin C, Perez JM, Peng D, Lyman WD, Chiu FC. Analysis of glial fibrillary acidic protein, neurofilament protein, actin and heat shock proteins in human fetal brain during the second trimester. Brain Res Dev Brain Res. 1996;91(1):1–10. 8821474 10.1016/0165-3806(95)00146-8

[97] Honig LS, Herrmann K, Shatz CJ. Developmental changes revealed by immunohistochemical markers in human cerebral cortex. Cereb Cortex. 1996;6(6):794–806. 8922336 10.1093/cercor/6.6.794

[98] Middeldorp J, Boer K, Sluijs JA, et al GFAPdelta in radial glia and subventricular zone progenitors in the developing human cortex. Development. 2010;137(2):313–321. 20040497 10.1242/dev.041632

[99] Tramontin AD, García-Verdugo JM, Lim DA, Alvarez-Buylla A. Postnatal development of radial glia and the ventricular zone (VZ): A continuum of the neural stem cell compartment. Cereb Cortex. 2003;13(6):580–587. 12764031 10.1093/cercor/13.6.580

[100] Sanai N, Tramontin AD, Quiñones-Hinojosa A, et al Unique astrocyte ribbon in adult human brain contains neural stem cells but lacks chain migration. Nature. 2004;427(6976):740–744. 14973487 10.1038/nature02301

[101] Morgan TE, Xie Z, Goldsmith S, et al The mosaic of brain glial hyperactivity during normal ageing and its attenuation by food restriction. Neuroscience. 1999;89(3):687–699. 10199605 10.1016/s0306-4522(98)00334-0

[102] Palmer AL, Ousman SS. Astrocytes and aging. Front Aging Neurosci. 2018;10:337, Accessed 7 December 2023. https://www.frontiersin.org/articles/10.3389/fnagi.2018.00337. 30416441 10.3389/fnagi.2018.00337 PMC6212515

[103] Nichols NR, Day JR, Laping NJ, Johnson SA, Finch CE. GFAP mRNA increases with age in rat and human brain. Neurobiol Aging. 1993;14(5):421–429. 8247224 10.1016/0197-4580(93)90100-p

[104] Messing A, Head MW, Galles K, Galbreath EJ, Goldman JE, Brenner M. Fatal encephalopathy with astrocyte inclusions in GFAP transgenic mice. Am J Pathol. 1998;152(2):391–398. 9466565 PMC1857948

[105] Herndon RM, Rubinstein LJ, Freeman JM, Mathieson G. Light and electron microscopic observations on Rosenthal fibers in Alexander’s disease and in multiple sclerosis. J Neuropathol Exp Neurol. 1970;29(4):524–551. 5471920 10.1097/00005072-197010000-00002

[106] Lowe J, Blanchard A, Morrell K, et al Ubiquitin is a common factor in intermediate filament inclusion bodies of diverse type in man, including those of Parkinson’s disease, Pick’s disease, and Alzheimer’s disease, as well as Rosenthal fibres in cerebellar astrocytomas, cytoplasmic bodies in muscle, and mallory bodies in alcoholic liver disease. J Pathol. 1988;155(1):9–15. 2837558 10.1002/path.1711550105

[107] Anderson MA, Burda JE, Ren Y, et al Astrocyte scar formation aids central nervous system axon regeneration. Nature. 2016;532(7598):195–200. 27027288 10.1038/nature17623 PMC5243141

[108] Pekny M, Pekna M. Reactive gliosis in the pathogenesis of CNS diseases. Biochim Biophys Acta. 2016;1862(3):483–491. 26655603 10.1016/j.bbadis.2015.11.014

[109] Kumar A, Fontana IC, Nordberg A. Reactive astrogliosis: A friend or foe in the pathogenesis of Alzheimer’s disease. J Neurochem. 2023;164(3):309–324. 34931315 10.1111/jnc.15565

[110] Jack CR, Knopman DS, Jagust WJ, et al Hypothetical model of dynamic biomarkers of the Alzheimer’s pathological cascade. Lancet Neurol. 2010;9(1):119–128. 20083042 10.1016/S1474-4422(09)70299-6 PMC2819840

[111] Yu G, Zhang Y, Ning B. Reactive astrocytes in central nervous system injury: Subgroup and potential therapy. Front Cell Neurosci. 2021;15:792764, Accessed 20 November 2023. https://www.frontiersin.org/articles/10.3389/fncel.2021.792764. 35002629 10.3389/fncel.2021.792764 PMC8733560

[112] Ross GW, O’Callaghan JP, Sharp DS, et al Quantification of regional glial fibrillary acidic protein levels in Alzheimer’s disease. Acta Neurol Scand. 2003;107(5):318–323. 12713522 10.1034/j.1600-0404.2003.02098.x

[113] Sajja VSSS, Hlavac N, VandeVord PJ. Role of glia in memory deficits following traumatic brain injury: Biomarkers of glia dysfunction. Front Integr Neurosci. 2016;10:7. 26973475 10.3389/fnint.2016.00007 PMC4770450

[114] Wyss-Coray T, Loike JD, Brionne TC, et al Adult mouse astrocytes degrade amyloid-beta in vitro and in situ. Nat Med. 2003;9(4):453–457. 12612547 10.1038/nm838

[115] Perez-Nievas BG, Serrano-Pozo A. Deciphering the astrocyte reaction in Alzheimer’s disease. Front Aging Neurosci. 2018;10:114, Accessed 11 December 2023. https://www.frontiersin.org/articles/10.3389/fnagi.2018.00114. 29922147 10.3389/fnagi.2018.00114 PMC5996928

[116] Xu K, Malouf AT, Messing A, Silver J. Glial fibrillary acidic protein is necessary for mature astrocytes to react to beta-amyloid. Glia. 1999;25(4):390–403. 10028921 10.1002/(sici)1098-1136(19990215)25:4<390::aid-glia8>3.0.co;2-7

[117] Bellaver B, Povala G, Ferreira PCL, et al Astrocyte reactivity influences amyloid-β effects on tau pathology in preclinical Alzheimer’s disease. Nat Med. 2023;29(7):1775–1781. 37248300 10.1038/s41591-023-02380-x PMC10353939

[118] Chiarini A, Armato U, Gardenal E, Gui L, Dal Prà I. Amyloid β-exposed human astrocytes overproduce phospho-tau and overrelease it within exosomes, effects suppressed by calcilytic NPS 2143—Further implications for Alzheimer’s therapy. Front Neurosci. 2017;11:217, Accessed 11 December 2023. https://www.frontiersin.org/articles/10.3389/fnins.2017.00217. 28473749 10.3389/fnins.2017.00217 PMC5397492

[119] Gulisano W, Maugeri D, Baltrons MA, et al Role of amyloid-β and tau proteins in Alzheimer’s disease: Confuting the amyloid cascade. J Alzheimers Dis. 2018;64(Suppl 1):S611–S631. 29865055 10.3233/JAD-179935 PMC8371153

[120] Gaikwad S, Puangmalai N, Bittar A, et al Tau oligomer induced HMGB1 release contributes to cellular senescence and neuropathology linked to Alzheimer’s disease and frontotemporal dementia. Cell Rep. 2021;36(3):109419. 34289368 10.1016/j.celrep.2021.109419 PMC8341760

[121] Milton NGN . Role of hydrogen peroxide in the aetiology of Alzheimer’s disease: Implications for treatment. Drugs Aging. 2004;21(2):81–100. 14960126 10.2165/00002512-200421020-00002

[122] Targa Dias Anastacio H, Matosin N, Ooi L. Neuronal hyperexcitability in Alzheimer’s disease: What are the drivers behind this aberrant phenotype? Transl Psychiatry. 2022;12(1):1–14. 35732622 10.1038/s41398-022-02024-7 PMC9217953

[123] Kwon HS, Koh SH. Neuroinflammation in neurodegenerative disorders: The roles of microglia and astrocytes. Transl Neurodegener. 2020;9(1):42. 33239064 10.1186/s40035-020-00221-2 PMC7689983

[124] Brawek B, Garaschuk O. Network-wide dysregulation of calcium homeostasis in Alzheimer’s disease. Cell Tissue Res. 2014;357(2):427–438. 24553999 10.1007/s00441-014-1798-8

[125] Sama DM, Norris CM. Calcium dysregulation and neuroinflammation: Discrete and integrated mechanisms for age-related synaptic dysfunction. Ageing Res Rev. 2013;12(4):982–995. 23751484 10.1016/j.arr.2013.05.008 PMC3834216

[126] Sompol P, Furman JL, Pleiss MM, et al Calcineurin/NFAT signaling in activated astrocytes drives network hyperexcitability in Aβ-bearing mice. J Neurosci. 2017;37(25):6132–6148. 28559377 10.1523/JNEUROSCI.0877-17.2017 PMC5481945

[127] Hase Y, Horsburgh K, Ihara M, Kalaria RN. White matter degeneration in vascular and other ageing-related dementias. J Neurochem. 2018;144(5):617–633. 29210074 10.1111/jnc.14271

[128] Yang Z, Wang KKW. Glial fibrillary acidic protein: From intermediate filament assembly and gliosis to neurobiomarker. Trends Neurosci. 2015;38(6):364–374. 25975510 10.1016/j.tins.2015.04.003 PMC4559283

[129] Giannoni P, Badaut J, Dargazanli C, et al The pericyte-glia interface at the blood-brain barrier. Clin Sci (Lond). 2018;132(3):361–374. 29439117 10.1042/CS20171634

[130] Blood–brain barrier breakdown is an early biomarker of human cognitive dysfunction | Nature Medicine. Accessed 19 July 2024. https://www.nature.com/articles/s41591-018-0297-y 10.1038/s41591-018-0297-y PMC6367058 30643288

[131] Bell RD, Winkler EA, Singh I, et al Apolipoprotein E controls cerebrovascular integrity via cyclophilin A. Nature. 2012;485(7399):512–516. 22622580 10.1038/nature11087 PMC4047116

[132] Yamazaki Y, Shinohara M, Shinohara M, et al Selective loss of cortical endothelial tight junction proteins during Alzheimer’s disease progression. Brain. 2019;142(4):1077–1092. 30770921 10.1093/brain/awz011 PMC6439325

[133] Greenberg SM, Bacskai BJ, Hernandez-Guillamon M, Pruzin J, Sperling R, van Veluw SJ. Cerebral amyloid angiopathy and Alzheimer disease—One peptide, two pathways. Nat Rev Neurol. 2020;16(1):30–42. 31827267 10.1038/s41582-019-0281-2 PMC7268202

[134] Iliff JJ, Wang M, Liao Y, et al A paravascular pathway facilitates CSF flow through the brain parenchyma and the clearance of interstitial solutes, including amyloid β. Sci Transl Med. 2012;4(147):147ra111. 10.1126/scitranslmed.3003748 PMC3551275 22896675

[135] Harrison IF, Ismail O, Machhada A, et al Impaired glymphatic function and clearance of tau in an Alzheimer’s disease model. Brain. 2020;143(8):2576–2593. 32705145 10.1093/brain/awaa179 PMC7447521

[136] Simon MJ, Iliff JJ. Regulation of cerebrospinal fluid (CSF) flow in neurodegenerative, neurovascular and neuroinflammatory disease. Biochim Biophys Acta. 2016;1862(3):442–451. 26499397 10.1016/j.bbadis.2015.10.014 PMC4755861

[137] Ishida K, Yamada K, Nishiyama R, et al Glymphatic system clears extracellular tau and protects from tau aggregation and neurodegeneration. J Exp Med. 2022;219(3):e20211275. 35212707 10.1084/jem.20211275 PMC8932543

[138] Singh R, Rai S, Bharti PS, et al Circulating small extracellular vesicles in Alzheimer’s disease: A case–control study of neuro-inflammation and synaptic dysfunction. BMC Med. 2024;22(1):254. 38902659 10.1186/s12916-024-03475-z PMC11188177

[139] Missler U, Wiesmann M, Wittmann G, Magerkurth O, Hagenström H. Measurement of glial fibrillary acidic protein in human blood: Analytical method and preliminary clinical results. Clin Chem. 1999;45(1):138–141. 9895354

[140] Mayer CA, Brunkhorst R, Niessner M, Pfeilschifter W, Steinmetz H, Foerch C. Blood levels of glial fibrillary acidic protein (GFAP) in patients with neurological diseases. PLoS ONE. 2013;8(4):e62101. 23626774 10.1371/journal.pone.0062101 PMC3633915

[141] Commissioner Office of the FDA authorizes marketing of first blood test to aid in the evaluation of concussion in adults. FDA. March 24, 2020. Accessed 22 November 2023. https://www.fda.gov/news-events/press-announcements/fda-authorizes-marketing-first-blood-test-aid-evaluation-concussion-adults

[142] Baiardi S, Quadalti C, Mammana A, et al Diagnostic value of plasma p-Tau181, NfL, and GFAP in a clinical setting cohort of prevalent neurodegenerative dementias. Alzheimers Res Ther. 2022;14(1):153. 36221099 10.1186/s13195-022-01093-6 PMC9555092

[143] Therriault J, Servaes S, Tissot C, et al Equivalence of plasma p-Tau217 with cerebrospinal fluid in the diagnosis of Alzheimer’s disease. Alzheimers Dement. 2023;19(11):4967–4977. 37078495 10.1002/alz.13026 PMC10587362

[144] Martínez-Dubarbie F, Guerra-Ruiz A, López-García S, et al Accuracy of plasma Aβ40, Aβ42, and p-Tau181 to detect CSF Alzheimer’s pathological changes in cognitively unimpaired subjects using the Lumipulse automated platform. Alzheimers Res Ther. 2023;15(1):163. 37784138 10.1186/s13195-023-01319-1 PMC10544460

[145] Nagele RG, D’Andrea MR, Lee H, Venkataraman V, Wang HY. Astrocytes accumulate A beta 42 and give rise to astrocytic amyloid plaques in Alzheimer disease brains. Brain Res. 2003;971(2):197–209. 12706236 10.1016/s0006-8993(03)02361-8

[146] Rasing I, Voigt S, Koemans EA, et al Serum and cerebrospinal fluid neurofilament light chain and glial fibrillary acid protein levels in early and advanced stages of cerebral amyloid Angiopathy. Alzheimers Res Ther. 2024;16(1):86. 38654326 10.1186/s13195-024-01457-0 PMC11036675

[147] Chatterjee P, Pedrini S, Stoops E, et al Plasma glial fibrillary acidic protein is elevated in cognitively normal older adults at risk of Alzheimer’s disease. Transl Psychiatry. 2021;11(1):1–10. 33431793 10.1038/s41398-020-01137-1 PMC7801513

[148] Thambisetty M, Lovestone S. Blood-based biomarkers of Alzheimer’s disease: Challenging but feasible. Biomark Med. 2010;4(1):65–79. 20387303 10.2217/bmm.09.84 PMC2863057

[149] Tijms BM, Vromen EM, Mjaavatten O, et al Cerebrospinal fluid proteomics in patients with Alzheimer’s disease reveals five molecular subtypes with distinct genetic risk profiles. Nat Aging. 2024;4(1):33–47. 38195725 10.1038/s43587-023-00550-7 PMC10798889

[150] Schindler SE, Bateman RJ. Combining blood-based biomarkers to predict risk for Alzheimer’s disease dementia. Nat Aging. 2021;1(1):26–28. 37117995 10.1038/s43587-020-00008-0

[151] Coppens S, Lehmann S, Hopley C, Hirtz C. Neurofilament-light, a promising biomarker: Analytical, metrological and clinical challenges. Int J Mol Sci. 2023;24(14):11624. 37511382 10.3390/ijms241411624 PMC10380627

[152] Mielke MM, Hagen CE, Xu J, et al Plasma phospho-tau181 increases with Alzheimer’s disease clinical severity and is associated with tau- and amyloid-positron emission tomography. Alzheimers Dement. 2018;14(8):989–997. 29626426 10.1016/j.jalz.2018.02.013 PMC6097897

[153] Fowler CJ, Stoops E, Rainey-Smith SR, et al Plasma p-Tau181/Aβ_1-42_ ratio predicts Aβ-PET status and correlates with CSF-p-Tau181/Aβ_1-42_ and future cognitive decline. Alzheimers Dement (Amst). 2022;14(1):e12375. 36447478 10.1002/dad2.12375 PMC9695763

[154] Verberk IMW, Thijssen E, Koelewijn J, et al Combination of plasma amyloid beta(1-42/1-40) and glial fibrillary acidic protein strongly associates with cerebral amyloid pathology. Alzheimers Res Ther. 2020;12(1):118. 32988409 10.1186/s13195-020-00682-7 PMC7523295

[155] Yang Z, Sreenivasan K, Toledano Strom EN, et al Clinical and biological relevance of glial fibrillary acidic protein in Alzheimer’s disease. Alzheimers Res Ther. 2023;15(1):190. 37924152 10.1186/s13195-023-01340-4 PMC10623866

[156] Sarto J, Ruiz-García R, Guillén N, et al Diagnostic performance and clinical applicability of blood-based biomarkers in a prospective memory clinic cohort. Neurology. 2023;100(8):e860–e873. 36450604 10.1212/WNL.0000000000201597 PMC9984216

[157] Stocker H, Beyer L, Trares K, et al Association of kidney function with development of Alzheimer disease and other dementias and dementia-related blood biomarkers. JAMA Netw Open. 2023;6(1):e2252387. 36692879 10.1001/jamanetworkopen.2022.52387 PMC10408272

